# MBOAT7-driven lysophosphatidylinositol acylation in adipocytes contributes to systemic glucose homeostasis

**DOI:** 10.1016/j.jlr.2023.100349

**Published:** 2023-02-18

**Authors:** William J. Massey, Venkateshwari Varadharajan, Rakhee Banerjee, Amanda L. Brown, Anthony J. Horak, Rachel C. Hohe, Bryan M. Jung, Yunguang Qiu, E. Ricky Chan, Calvin Pan, Renliang Zhang, Daniela S. Allende, Belinda Willard, Feixiong Cheng, Aldons J. Lusis, J. Mark Brown

**Affiliations:** 1 Department of Cardiovascular and Metabolic Sciences, Lerner Research Institute, Cleveland Clinic, Cleveland, OH, USA; 2 Center for Microbiome and Human Health, Lerner Research Institute, Cleveland Clinic, Cleveland, OH, USA; 3 Genomic Medicine Institute, Lerner Research Institute, Cleveland Clinic, Cleveland, OH, USA; 4 Institute for Computational Biology, Case Western Reserve University, Cleveland, OH, USA; 5 Departments of Medicine, Microbiology, and Human Genetics, University of California Los Angeles, Los Angeles, CA, USA; 6 Proteomics and Metabolomics Core, Lerner Research Institute, Cleveland Clinic, Cleveland, OH, USA; 7 Department of Anatomical Pathology, Cleveland Clinic, Cleveland, OH, USA

**Keywords:** non-alcoholic fatty liver disease, obesity, metabolism, diabetes, hyperinsulinemia, systemic insulin resistance, arachidonic acid, acyltransferase, phosphatidylinositol biosynthesis, hepatocytes

## Abstract

We previously demonstrated that antisense oligonucleotide-mediated knockdown of Mboat7, the gene encoding membrane bound O-acyltransferase 7, in the liver and adipose tissue of mice promoted high fat diet-induced hepatic steatosis, hyperinsulinemia, and systemic insulin resistance. Thereafter, other groups showed that hepatocyte-specific genetic deletion of Mboat7 promoted striking fatty liver and NAFLD progression in mice but does not alter insulin sensitivity, suggesting the potential for cell autonomous roles. Here, we show that MBOAT7 function in adipocytes contributes to diet-induced metabolic disturbances including hyperinsulinemia and systemic insulin resistance. We generated Mboat7 floxed mice and created hepatocyte- and adipocyte-specific Mboat7 knockout mice using Cre-recombinase mice under the control of the albumin and adiponectin promoter, respectively. Here, we show that MBOAT7 function in adipocytes contributes to diet-induced metabolic disturbances including hyperinsulinemia and systemic insulin resistance. The expression of Mboat7 in white adipose tissue closely correlates with diet-induced obesity across a panel of ∼100 inbred strains of mice fed a high fat/high sucrose diet. Moreover, we found that adipocyte-specific genetic deletion of Mboat7 is sufficient to promote hyperinsulinemia, systemic insulin resistance, and mild fatty liver. Unlike in the liver, where Mboat7 plays a relatively minor role in maintaining arachidonic acid-containing PI pools, Mboat7 is the major source of arachidonic acid-containing PI pools in adipose tissue. Our data demonstrate that MBOAT7 is a critical regulator of adipose tissue PI homeostasis, and adipocyte MBOAT7-driven PI biosynthesis is closely linked to hyperinsulinemia and insulin resistance in mice.

The rising prevalence of obesity closely parallels the rise of nonalcoholic fatty liver disease (NAFLD), which is a leading cause of liver disease mortality in developed countries (1, 2, 3, 4). Because of this rapidly growing health crisis, there is an increasing need for mechanistic insight to allow a path for the rational design of new therapeutic strategies. One important clue in NAFLD research has recently emerged where multiple genome wide association studies identified the common rs641738 SNP located close to the lysophosphatidylinositol (LPI)-acylating enzyme, membrane bound O-acyltransferase 7 (MBOAT7), as a risk allele. The rs641738 (C>T) SNP is associated with increased susceptibility to NAFLD (5, 6, 7), liver diseases of alcoholic and viral etiologies (8, 9, 10), and other complex metabolic diseases (11, 12). Using an antisense oligonucleotide (ASO)-mediated approach, we recently showed that *Mboat7* knockdown exacerbated high fat diet (HFD)-induced hepatic steatosis and inflammation, hyperinsulinemia, and insulin resistance (13). It is important to note that we also showed that genetic deletion of the neighboring gene transmembrane channel like 4 did not promote NAFLD (13).

Very similar findings were reported by Meroni *et al.* (14) using a morpholino oligonucleotide (MPO)-driven knockdown approach, further demonstrating that *Mboat7* loss-of-function promoted fatty liver and importantly showed that *Mboat7* expression is suppressed by insulin. More recently, several independent laboratories have shown that hepatocyte-specific deletion of *Mboat7* (*Mboat7*^HSKO^) worsens hepatic steatosis, inflammation, and fibrosis, but unlike the oligonucleotide-based knockdown, does not impact insulin or glucose homeostasis (15, 16, 17). Given the disparate results between the results of published ASO and MPO knockdown experiments (13, 14) and those of these recent studies of *Mboat7*^HSKO^ (15, 16, 17), we hypothesized that the function of MBOAT7 in adipocytes may be critically important in regulating circulating insulin levels and action in target tissues. It is well appreciated that ASOs can effectively suppress target gene expression in adipocytes (13, 18) and adipose tissue plays a critically important role in the progression of NAFLD and systemic glucose homeostasis. To better understand the tissue-specific roles of MBOAT7, we generated *Mboat7* adipocyte-specific KO (*Mboat7*^ASKO^) and *Mboat7* hepatocyte-specific KO (*Mboat7*^HSKO^) mice to understand the cell-autonomous contributions of *Mboat7* to HFD-induced metabolic disturbances. Here, we find that adipocyte *Mboat7* contributes less so to hepatic steatosis and injury than hepatocyte *Mboat7* but instead plays a critically important role in regulating local adipose tissue LPI/PI homeostasis, hyperinsulinemia, and systemic insulin sensitivity.

## Materials and methods

### Mice and experimental diets

To generate conditional *Mboat7* KO mice, we obtained “KO first” (Mboat7^tm1a(KOMP)Wtsi^) mice from Dr Philip Hawkins (19) and crossed these mice with mice transgenically expressing FLP recombinase to remove the NEO cassette resulting in a conditional *Mboat7* floxed allele. The FLP transgene was then bred out of the line and resulting *Mboat7*^flox/WT^ mice were subsequently bred with transgenic mice expressing Cre recombinase under either the adiponectin promoter/enhancer (for adipocyte-specific deletion) or under the albumin promoter (for hepatocyte-specific deletion). Importantly, each substrain of mice was backcrossed >10 generations into the C57BL/6J background to produce congenic lines, and confirmation of sufficient backcrossing into the C57BL/6J background was confirmed by mouse genome SNP scanning at the Jackson Laboratory (Bar Harbor, ME). For all experiments, age-matched animals were put on either standard rodent chow (Teklad 2918) or 60% kcal fat HFD (Research Diets D12492) for up to 20 weeks. Glucose and insulin tolerance tests (ITTs) were performed as previously described (20, 21, 22, 23, 24, 25) in mice following 12 and 14 weeks of concurrent chow and HFD-feeding, respectively. Quantitation of lean and fat mass were done using an EchoMRITM-130 Body Composition Analyzer (EchoMRI International). All mice were maintained in an Association for the Assessment and Accreditation of Laboratory Animal Care, International-approved animal facility, and all experimental protocols were approved by the IACUC of the Cleveland Clinic (IACUC protocols # 2018-2053 and # 00002499).

### Standardized necropsy conditions

To keep results consistent, the experimental mice were fasted for 4 h (from 09:00 to 13:00) prior to necropsy for 20 weeks diet study and 16 weeks HFD study. For 12 weeks HFD study with fasting-refeeding, mice were fasted overnight (16:00–08:00), then bled via submandibular bleed and refed HFD for 3 h (until 11:00). At necropsy, all mice were terminally anesthetized with ketamine/xylazine (100–160 mg/kg ketamine-20–32 mg/kg xylazine) and a midline laparotomy was performed. Blood was collected by heart puncture. Following blood collection, a whole-body perfusion was conducted by puncturing the right atria and slowly delivering 10 ml of saline into the left ventricle of the heart to remove blood from tissues. Tissues were collected and immediately snap frozen in liquid nitrogen for subsequent biochemical analysis or fixed for morphological analysis. Various adipose depots were defined by their anatomical location. Gonadal white adipose tissue (gWAT) was dissected from the area surrounding the testes (males) or fallopian tubes (females), subcutaneous white adipose tissue was dissected from the subcutaneous surface of the overlying inguinal skin, retroperitoneal adipose tissue was collected from the retroperitoneum (beneath the kidneys), mesenteric adipose tissue was collected by dissecting away the pancreas and then careful dissection of fat from the remaining intestinal tissue, and brown adipose tissue (BAT) was carefully dissected from the subscapular space to remove overlying white adipose tissue (WAT).

### Hyperinsulinemic-euglycemic clamp

All procedures required for the hyperinsulinemic–euglycemic clamp were approved by the Vanderbilt University IACUC and performed at the Vanderbilt Mouse Metabolic Phenotyping Center. For these studies, age-matched *Mboat7*^flox/flox^ and *Mboat7*^ASKO^ mice were maintained on HFD for 11–12 weeks. Thereafter, while being maintained on HFD, catheters were implanted into a carotid artery and a jugular vein of mice for sampling and infusions respectively, 5 days before the study as described previously (26). Insulin clamps were performed on mice fasted for 5 h using a modification of the method described by Ayala *et al.* (27). [3-3H]-glucose was primed (1.5 μCi) and continuously infused for a 90 min equilibration and basal sampling periods (0.075 μCi/min). [3-3H]-glucose was mixed with the nonradioactive glucose infusate (infusate specific-activity of 0.5 μCi/mg) during the 2 h clamp period. Arterial glucose was clamped using a variable rate of glucose (plus trace [3-3H]-glucose) infusion, which was adjusted based on the measurement of blood glucose at 10 min intervals. By mixing radioactive glucose with the nonradioactive glucose infused during a clamp, deviations in arterial glucose-specific activity are minimized and steady state conditions are achieved. The calculation of glucose kinetics is therefore more robust (28). Baseline blood or plasma variables were calculated as the mean of values obtained in blood samples collected at −15 and −5 min. At time zero, insulin infusion (4 mU/kg body weight/min) was started and continued for 120 min. Mice received heparinized saline-washed erythrocytes from donors at 5 μl/min to prevent a fall in hematocrit. Blood was taken from 80 to 120 min for the determination of [3-3H]-glucose. Clamp insulin was determined at t = 100 and 120 min. At 120 min, 13 μCi of 2[14C]deoxyglucose ([14C]2DG) was administered as an intravenous bolus. Blood was taken from 2 to 25 min for determination of [14C]2DG. After the last sample, mice were anesthetized and tissues were freeze-clamped for further analysis. Radioactivity of [3-3H]-glucose and [14C]2DG in plasma samples and [14C]2DG-6-phosphate in tissue samples were determined by liquid scintillation counting. Glucose appearance (Ra) and disappearance (Rd) rates were determined using steady-state equations (29). Endogenous glucose appearance (endoRa) was determined by subtracting the glucose infusion rate from total Ra. The glucose metabolic index (Rg) was calculated as previously described (30).

### Histological analysis and imaging

H&E staining of paraffin-embedded liver sections was performed as previously described (21, 22, 23, 24, 25). Histopathologic evaluation was scored in a blinded fashion by a board-certified pathologist with expertise in gastrointestinal/liver pathology (Daniela S. Allende–Cleveland Clinic). H&E slides were scanned using a Leica Aperio AT2 Slide Scanner (Leica Microsystems, GmbH, Wetzlar, Germany; https://www.leicabiosystems.com/us/digital-pathology/manage/aperio-imagescope/) and images were processed using ImageScope (Aperio, Software Version 12.1).

### Quantification of adipose tissue immune cell populations by flow cytometry

After 16 weeks of HFD-feeding, gWAT was excised, washed with 1× PBS, and immediately placed into RPMI with 1 mg/ml Type II Collagenase (Sigma Aldrich, St. Louis, MO) for 30 min at 37°C with gentle agitation. Digested clumps of tissue were pressed through a 100 μm strainer and washed with 1× PBS. Cells were centrifuged at 500 *g* for 10 min; supernatant was aspirated. Cell pellet containing the stromal vascular fraction was resuspended in ammonium-chloride-potassium Lysing Buffer (Life Technologies, Grandstand, NY) for 5 min at room temperature. Cells were washed with 1× PBS and centrifuged at 300 *g* for 10 min. For flow cytometry, cells were resuspended in freshly prepared fluorescence-activated cell sorting (FACS) buffer (1× PBS, 3% FBS) and aliquoted into 96-well plates. Cells were centrifuged at 830 *g* for 4 min, resuspended in 50 μl FACS buffer containing 0.5 μg anti-mouse CD16/CD32 mAb 2.4G2 (Mouse BD Fc Block, BD Pharmingen, San Diego, CA), and incubated for 15 min 4°C. After blocking, cells were stained with a fluorochrome-conjugated antibody panel (antibodies described in the key resource table) for 30 min at 4°C in the dark. Cells were washed and centrifuged at 830 *g* for 4 min twice with FACS buffer. Stained cells are resuspended in 200 μl of 1% paraformaldehyde and kept in the dark at 4°C overnight. Stained cells were centrifuged at 830 *g* for 5 min. Stained cells were resuspended in 300 μl of FACS buffer and data was collected on a Cytek Aurora full spectrum (365–829 nm range) cytometer using SpectroFlo® software (Cytek® Biosciences, Fremont, CA; https://cytekbio.com/pages/spectro-flo). Data collected on the Aurora were analyzed using FlowJo software (Tree Star, Inc., Ashland, OR; https://www.flowjo.com/). Gating strategies are shown in supplemental Fig. S10.

### Immunoblotting

Whole tissue homogenates were made from tissues in a modified RIPA buffer as previously described (20, 21, 22, 23, 24, 25) that was supplemented with an additional 2% (w/v) sodium dodecylsulfate, and protein was quantified using the BCA assay (Pierce). 10 μg of protein was separated by 4–12% SDS-PAGE, transferred to a PVDF membrane, and proteins were detected after incubation with specific antibodies as previously described (20, 21, 22, 23, 24, 25).

### RNA isolation and bulk RNA sequencing

gWAT RNA was isolated using Trizol-Chloroform extraction, gDNA removal by eliminator spin column (Qiagen), and isopropanol precipitation followed by ethanol clean-up. RNA quality was confirmed by Bioanalyzer (Agilent). RNA-SEQ libraries were generated using the Illumina mRNA TruSEQ Directional library kit and sequenced using an Illumina HiSEQ4000 (both according to the Manufacturer’s instructions). RNA sequencing was performed by the University of Chicago Genomics Facility. Raw sequencing data in the form of FASTQ files were transferred to and analyzed by the Bioinformatics Core at Case Western Reserve University. FASTQ files were trimmed for quality and adapter sequences using TrimGalore! (version 0.6.5 Babraham Institute, https://github.com/FelixKrueger/TrimGalore), a wrapper script for CutAdapt and FastQC. Reads passing quality control were aligned to the mm10 mouse reference genome using STARAligner (31) (version 2.5.3a) and guided using the GENCODE gene annotation. Aligned reads were analyzed for differential gene expression using Cufflinks (32) (version 2.2.1) which reports the fragments per kilobase of exon per million fragments mapped for each gene. Significant genes were identified using a significance cutoff of q-value <0.05 (FDR) and used as input for downstream analysis. RNA sequencing data can be accessed at GEO Profiles at the NCBI: GSE203414 and full accession will be available upon acceptance of this work. In addition, the RNA expression data can be found in supplemental Table S2 associated with the online version of this article.

### Plasma and liver biochemistries

To determine the level of hepatic injury in mice fed HFD, plasma was used to analyze alanine aminotransferase levels using enzymatic assays as previously described (13). Extraction of liver lipids and quantification of total plasma and hepatic triglycerides, total cholesterol, and free cholesterol was conducted using enzymatic assays as described previously (21, 22, 23, 24, 25). Esterified cholesterol was calculated by subtracting free cholesterol from total cholesterol.

### Targeted quantification of phosphatidylinositol (PI) and lysophosphatidylinositol lipids

Quantitation of LPI and PI species was performed as previously described (13). Briefly, LPI and PI standards (LPI-16:0, LPI-18:0, LPI-18:1, LPI-20:4, PI-38:4) and the two internal standards (LPI-17:1-d31, PI-34:1-d31) were purchased from Avanti Polar Lipids. HPLC grade water, methanol, and acetonitrile were purchased from Thermo Fisher Scientific. Standard LPI and PI species at concentrations of 0, 5, 20, 100, 500, and 2,000 ng/ml were prepared in 90% methanol containing 2 internal standards at the concentration of 500 ng/ml. The volume of 5 μl was injected into the Shimadzu LCMS-8050 for generating the internal standard calibration curves. A triple quadrupole mass spectrometer (Thermo Fisher Scientific Quantiva, Waltham, MA) was used for analysis of LPI and PI species. The mass spectrometer was coupled to the outlet of an ultra high performance liquid chromatography system (Vanquish, Thermo Fisher Scientific, Waltham, MA), including an auto sampler with refrigerated sample compartment and inline vacuum degasser. The HPLC eluent was directly injected into the triple quadrupole mass spectrometer and the analytes were ionized at ESI negative mode. Analytes were quantified using Selected Reaction Monitoring and the Selected Reaction Monitoring transitions (m/z) were 571 → 255 for LPI-16:0, 599 → 283 for LPI-18:0, 597 → 281 for LPI-18:1, 619 → 303 for LPI-20:4, 885 → 241 for PI-38:4, 583 → 267 for internal standard LPI-17:1, and 866 → 281 for internal standard PI-34:1-d31. Xcalibur software (https://www.thermofisher.com/order/catalog/product/OPTON-30965) was used to get the peak area for both the internal standards and LPI and PI species. The internal standard calibration curves were used to calculate the concentration of LPI and PI species in the samples.

### Adipose tissue phosphoproteomic and pathway analyses to examine LPI-induced signaling events in WAT

The goal of this experiment was to unbiasedly identify LPI-responsive signaling events in mouse WAT after an acute exposure (15 min) to a physiological level of LPI. Optimization of in vivo LPI dosing was previously described by Helsley *et al.* 2019 (13). Briefly, mice were fasted for 4 h between the hours of 09:00–13:00, then we delivered 50 μl of either sterile saline or 400 μM (20 nmol, 12.3 μg) 18:1 LPI intraperitoneally. After 10 min, mice were anesthetized and tissue was dissected as described above. Adipose tissue samples were homogenized, the protein was precipitated with acetone, and the protein concentration was measured. A total of 350 μg of protein from each sample was digested with trypsin and the resulting tryptic peptides were subjected to phosphoserine and phosphothreonine enrichment using the Thermo Fisher Scientific Phosphopeptide Enrichment (Thermo Fisher Scientific High Select™ A32992). The enrichment was performed based on the manufacturer’s instructions. The enriched peptide samples were dried then reconstituted in 25 μl 0.1% formic acid for LC-MS analysis. The LC-MS system was a Thermo Fisher Scientific Fusion Lumos mass spectrometer system. The HPLC column was a Dionex 15 cm × 75 μm id Acclaim Pepmap C18, 2 μm, 100 Å reversed-phase capillary chromatography column. Five μl volumes of the extract were injected and the peptides eluted from the column by an acetonitrile/0.1% formic acid gradient at a flow rate of 0.25 μl/min were introduced into the source of the mass spectrometer on-line. The microelectrospray ion source was operated at 1.9 kV. The digest was analyzed using the data-dependent multitask capability of the instrument acquiring full scan mass spectra to determine peptide molecular weights and product ion spectra to determine amino acid sequence in successive instrument scans. The LC-MS/MS data files were searched against the mouse UniProtKB databases using the program Proteome Discoverer 2.5 (https://www.thermofisher.com/us/en/home/industrial/mass-spectrometry/liquid-chromatography-mass-spectrometry-lc-ms/lc-ms-software/multi-omics-data-analysis/proteome-discoverer-software.html?gclid=EAIaIQobChMI9Kei3-rR_QIVYGxvBB3mnwJbEAAYASAAEgI-KvD_BwE&cid=E.23CMD.DL103.12911.01&ef_id=EAIaIQobChMI9Kei3-rR_QIVYGxvBB3mnwJbEAAYASAAEgI-KvD_BwE:G:s&s_kwcid=AL!3652!3!646724600131!p!!g!!proteome%20discoverer) considering Oxidation of Met, N-terminal Acetyl, S, T, and Y phosphorylation as a differential modification. A maximum of three missed cleavages were permitted. The peptide and protein false discovery rates were set to 0.01 using a target-decoy strategy. Phosphorylation sites were identified using ptmRS node in PD2.4. The relative abundance of the positively identified phosphopeptides was determined using the extracted ion intensities (Minora Feature Detection node) with Retention time alignment. All peptides were included in the quantitation, the peptide intensities were normalized to total peptide amount. Missing values were imputed in Perseus using a normal distribution. Kyoto Encyclopedia of Genes and Genomes (KEGG) enrichment analyses were performed to evaluate the biological relevance and functional pathways on the significant expressed genes of the LPI-related phosphoproteomics. The Entrez ID and official gene symbols were mapped based on GeneCards (http://www.genecards.org/). A total of 6,141 phosphopeptides were identified with 165 and 40 phosphopeptides determined to be |log_2_FoldChange| >0.5 different in the LPI and saline samples with a *P*-value <0.05 (two-tailed *t* test) in *Mboat7*^ASKO^ and *Mboat7*^flox/flox^ mice, respectively. The functional pathways were ranked by -log_10_FDR. Significant pathways were identified with FDR <0.05. The coverage of significant expressed genes among the total genes provided by KEGG were also calculated. All pathway analyses were performed by using Enrichr (33). Phosphoproteomics data have been deposited to the ProteomeXchange Consortium via the PRIDE (34) partner repository with the dataset identifier PXD039894 and can be found in supplemental Table S3 associated with the online version of this article.

### Statistical analyses, key reagents, and data availability

**Fig. 1:**
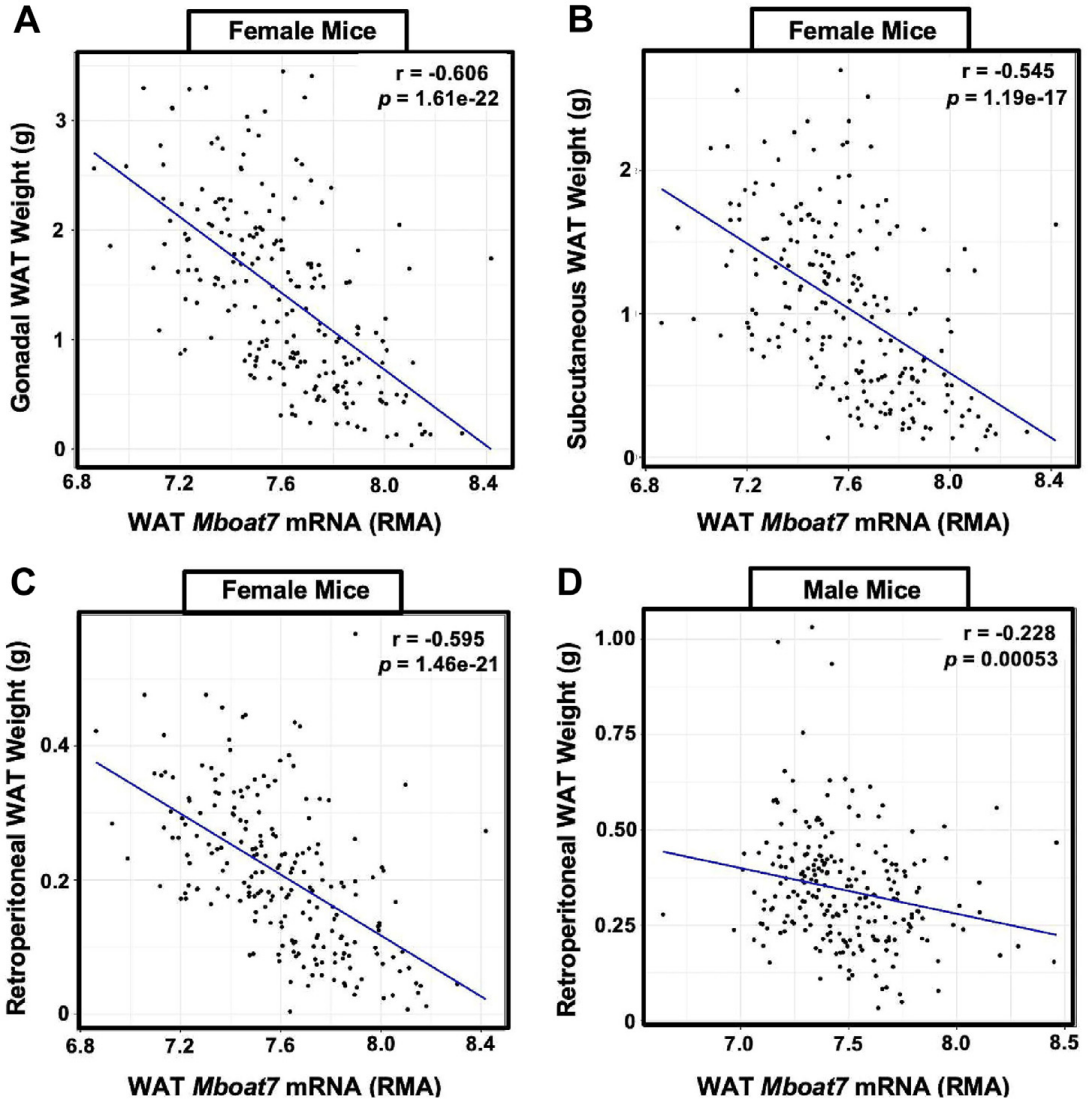
*Mboat7* Expression in White Adipose Tissue is Correlated with Adiposity in mice. We used a systems genetics approach to examine links between *Mboat7* expression and metabolic traits in mice from the hybrid mouse diversity panel (HMDP). To induce obesity, all mouse strains represented in the HMDP were fed an obesity-promoting high fat and high sucrose diet. Across the different strains in the HMDP, the expression (RMA, Robust Multi-Array Average) of *Mboat7* in adipose tissue has a strong negatively correlated with gonadal (A), subcutaneous (B), and retroperitoneal (C) white adipose tissue mass in females. There is a similar negative correlation in male (D) retroperitoneal adipose tissue. MBOAT7, membrane bound O-acyltransferase 7.

For the data shown in Fig. 1 [hybrid mouse diversity panel (HMDP)] correlations and associated *P*-values were calculated with the bi-weight midcorrelation, which is robust to outliers and associated *P*-value. Single comparisons between two groups were performed using two-tailed Student’s *t* tests with 95% confidence intervals. Comparisons involving multiple time points were assessed using an ordinary one-way ANOVA followed by Tukey’s multiple comparisons test, or using a two-way ANOVA followed by Tukey’s multiple comparisons test. All data presented as mean ± SEM. Values were considered significant at *P* < 0.05. ∗*P* < 0.05, ∗∗*P* < 0.01, ∗∗∗*P* < 0.001 and ∗∗∗∗*P* < 0.0001. For all other Fig. panels each experiment consisted of a minimum of two biological replicates and data are presented as mean ± SD except were noted in the Fig. legend. All data were analyzed using either one-, two-, or three-way ANOVA where appropriate, followed by either Tukey’s, Bonferroni’s, or Student’s *t*-tests for post hoc analysis. Differences were considered significant at *P* < 0.05. GraphPad Prism 9.4.0 (La Jolla, CA; https://www.graphpad.com/features) was used for data analysis. A list of key reagents can be found in supplemental Table S1. All materials, methods, and datasets included in this article are readily available upon request.

## Results

### Adipocyte-specific genetic deletion of *Mboat7* promotes mild fatty liver but profound hyperinsulinemia and insulin resistance

To understand the cell autonomous roles of *Mboat7* in HFD-driven metabolic disturbance we generated parallel colonies of either *Mboat7*^HSKO^ or *Mboat7*^ASKO^ mice and subjected them to HFD feeding. Our initial rationale to study the role of *Mboat7* outside of the liver came from studies where we used a systems genetics approach to examine tissue-specific links between *Mboat7* expression and adiposity in mice represented in the HMDP when challenged with an obesity-promoting HFD and high sucrose diet (35, 36). In our previous work, we found that *Mboat7* expression in the liver was only modestly correlated (r = −0.244, *P* = 0.01) with adiposity (13). However, here we show that *Mboat7* expression in WAT is strongly negatively correlated with gWAT, scWAT, and retroperitoneal WAT in female mice and retroperitoneal WAT in male mice (Fig. 1A–D). It is interesting to note that WAT *Mboat7* mRNA expression is not significantly correlated with adiposity measures in mice maintained on a standard rodent chow (data not shown), suggesting a role for WAT *Mboat7* in selectively shaping diet-driven metabolic disturbance.

**Fig. 2:**
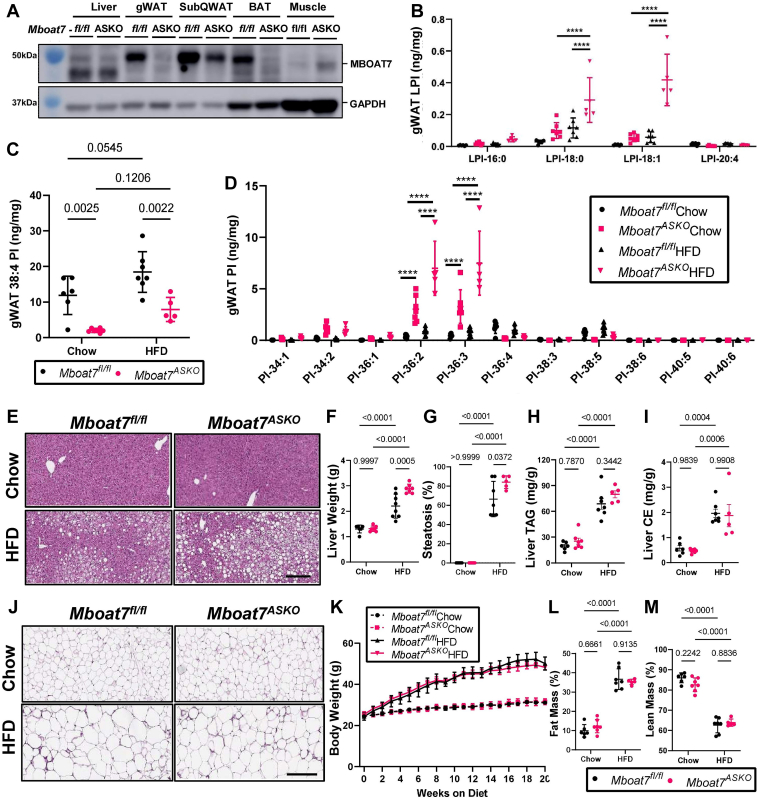
Adipocyte-Specific *Mboat7* Deletion (*Mboat7*^*ASKO*^) Promotes Mild Fatty Liver. Male control (*Mboat7*^*fl/fl*^) or adipocyte-specific Mboat7 knockout mice (*Mboat7*^*ASKO*^) were fed chow or high fat diet (HFD) for 20 weeks and metabolically phenotyped. A: Western blots from tissues collected from age-matched, chow fed *Mboat7*^*fl/fl*^ or *Mboat7*^*ASKO*^ mice. B–M: Gonadal white adipose tissue (gWAT), lysophosphatidylinositol (LPI), (B) and phosphatidylinositol (PI) species, including the MBOAT7 product PI-38:4 (C) and others (D) were quantified via LC-MSin *Mboat7*^*fl/fl*^ or *Mboat7*^*ASKO*^ mice were fed chow or high fat diet (HFD) for 20-week (n = 5–7; ∗∗∗∗*P* ≤ 0.0001; Two-way (C) or Three-way (B, D) ANOVA with Tukey’s *post hoc* test). E: Representative liver H&E stained sections. 10× magnification (scale bar = 200 μm). F: Liver weight measurements from *Mboat7*^*fl/fl*^ or *Mboat7*^*ASKO*^ mice fed Chow and HFD for 20 weeks (n = 6–8; Two-way ANOVA with Tukey’s *post hoc* test). G: Percent steatosis was quantified by a blinded pathologist (n = 5–7; Two-way ANOVA with Tukey’s *post hoc* test). Hepatic triglycerides (H) and hepatic esterified cholesterol (I) were measured enzymatically (n = 5–7; Two-way ANOVA with Tukey’s *post hoc* test). J: Representative gWAT H&E stained sections. 10× magnification (scale bar = 200 μm). K: Body weight was measured weekly. Percent fat (L) and % Lean (M) mass were determined via echo-MRI after 8 weeks of chow or HFD in *Mboat7*^*fl/fl*^ or *Mboat7*^*ASKO*^ mice (n = 5–7; Two-way ANOVA with Tukey’s *post hoc* test). All data are presented as mean ± S.D.

To generate congenic *Mboat7*^ASKO^ mice, we crossed mice harboring a post-FLP recombinase conditionally targeted *Mboat7* floxed allele (19) to transgenic mice expressing Cre recombinase under the adiponectin promoter/enhancer (37), and then backcrossed these mice >10 generations into the C57BL/6J background. This is important since the genetic background can significantly impact metabolic traits. Compared to age-matched, chow fed control mice (*Mboat7*^flox/flox^), *Mboat7*^ASKO^ mice had significantly reduced MBOAT7 protein expression in gWAT, scWAT, and subscapular BAT and a modest increase in skeletal muscle (Fig. 2A). It is important to note that liver MBOAT7 protein levels were not different in *Mboat7*^ASKO^ mice (Fig. 2A). However, the molecular weight of the major MBOAT7 protein isoform in the liver is slightly smaller than the major isoform in WAT and BAT (Fig. 2A). Adipocyte-specific deletion of *Mboat7* promotes marked reorganization of MBOAT7 substrate (LPI) and product (PI) lipids in gWAT (Fig. 2B–D). Particularly when challenged with a HFD, *Mboat7*^ASKO^ mice have significantly elevated levels of 16:0 LPI, 18:0 LPI, and 18:1 LPI lipids in gWAT (Fig. 2B). Reciprocally, the predominant enzymatic product of MBOAT7 (38:4 PI) is reduced by >60% in *Mboat7*^ASKO^ mice under both chow and HFD feeding conditions (Fig. 2C). Although less abundant, 36:4 PI and 38:5 PI lipids are also reduced in the gWAT of *Mboat7*^ASKO^ mice and there is a reciprocal increase in other more saturated species of PI (34:2 PI, 36:2 PI, and 36:3 PI) (Fig. 2D). It is important to note that the levels of LPI and PI lipids in the plasma and liver were unaffected in *Mboat7*^ASKO^ mice (supplemental Fig. S1), which is in contrast to what has been recently reported in *Mboat7*^HSKO^ mice where both liver and plasma LPI and PI homeostasis is altered (15, 16, 17). Collectively, these results demonstrate that MBOAT7 is a major regulator of the local LPI and PI lipidome in gWAT but unlike *Mboat7*^HSKO^ mice, adipocyte-specific deletion of *Mboat7* does not significantly alter circulating or liver LPI/PI homeostasis.

Next, we compared and contrasted HFD-driven metabolic phenotypes in *Mboat7*^ASKO^ and *Mboat7*^HSKO^ mice. In agreement with what has been previously reported by three independent groups (15, 16, 17), we also find that *Mboat7*^HSKO^ mice have significant reductions in 38:4 PI and elevation in its substrate LPI in the liver but not in gWAT (supplemental Fig. S2G–L). *Mboat7*^HSKO^ mice have profound hepatic steatosis and elevated alanine aminotransferase (supplemental Fig. S2C–F), confirming the concept that MBOAT7 activity in hepatocytes opposes hepatic steatosis and liver injury (15, 16, 17). Interestingly, adipocyte-specific deletion of *Mboat7* also promotes mild hepatomegaly and hepatic steatosis after 20 weeks of HFD feeding but not shorter durations (Fig. 2E–G and supplemental Fig. S3A). However, even after 20 weeks of HFD, the significantly increased hepatic triglyceride and cholesterol ester levels apparent in *Mboat7*^HSKO^ (supplemental Fig. S2E, (15, 16, 17)) are not seen in *Mboat7*^ASKO^ mice (Fig. 2H, I). It is important to note that upon HFD feeding total body weight and WAT histology are similar in *Mboat7*^HSKO^ when compared to their controls (supplemental Fig. S2B; and data not shown) as well as *Mboat7*^ASKO^ compared to their appropriate controls (Fig. 2J, K). We also found that, after 8 weeks of HFD feeding, there were no differences between *Mboat7*^ASKO^ and control mice in lean or fat mass as measured by EchoMRI (Fig. 2L, M). Collectively, these data suggest that MBOAT7 has clear cell autonomous roles in regulating circulating and tissue LPI and PI pools and MBOAT7-driven LPI acylation in hepatocytes is the primary driver of hepatic steatosis seen with MBOAT7 loss-of-function.

### Adipocyte-specific, but not hepatocyte-specific, deletion of *Mboat7* promotes hyperinsulinemia and insulin resistance

**Fig. 3:**
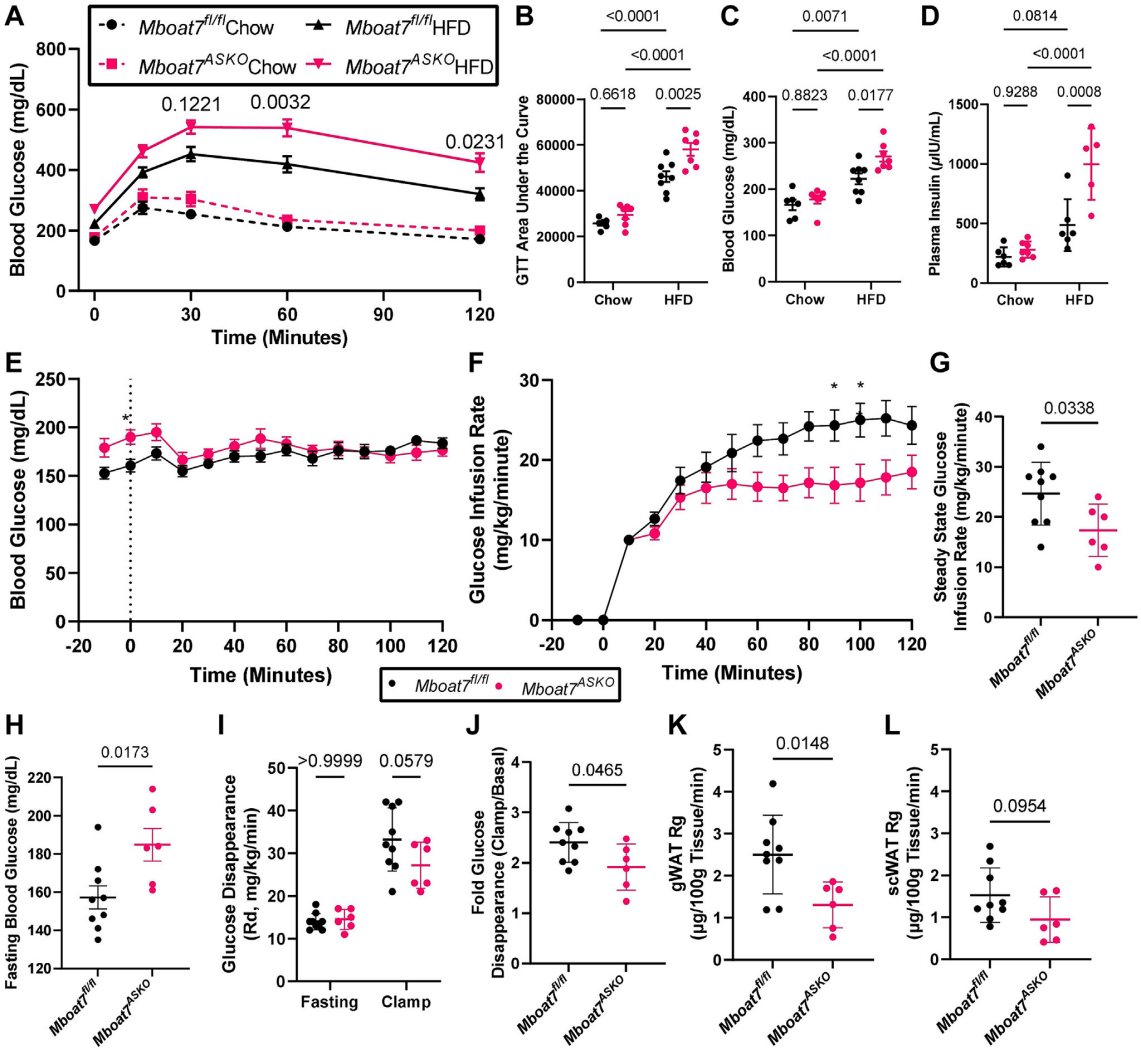
Adipocyte-Specific *Mboat7* Deletion (*Mboat7*^*ASKO*^) Promotes Glucose Intolerance, Hyperinsulinemia, and Peripheral Insulin Resistance. A–C: Male control (*Mboat7*^*fl/fl*^) or adipocyte-specific Mboat7 knockout mice (*Mboat7*^*ASKO*^) were fed a chow or HFD for 12 weeks and then underwent an intraperitoneal glucose tolerance test (GTT). A: Plasma glucose levels were measured (in duplicate or triplicate at each time point) throughout the GTT (n = 5–7; Three-way ANOVA with Tukey’s *post hoc* test). B: The area under the curve was calculated for each mouse throughout the GTT (n = 5–7; Two-way ANOVA with Tukey’s *post hoc* test). C: Fasting blood glucose was measured after a 4-h fast (time = 0 min for GTT) (n = 5–7; Two-way ANOVA with Tukey’s *post hoc* test). D: Fasting plasma insulin was measured in *Mboat7*^*fl/fl*^ or *Mboat7*^*ASKO*^ mice that were fed a chow or HFD for 20 weeks (n = 5–7; Two-way ANOVA with Tukey’s *post hoc* test). E–L: Male control (*Mboat7*^*fl/fl*^) or adipocyte-specific Mboat7 knockout mice (*Mboat7*^*ASKO*^) were fed HFD for 12–13 weeks, underwent surgery for catheterization of carotid artery and a jugular vein, and were subjected to euglycemic-hyperinsulinemic clamping. E: Plasma glucose levels were measured throughout the clamp (n = 6–9; Two-way ANOVA with Bonferroni’s post hoc test). F: Glucose infusion rates (GIRs) were measured throughout the clamp (n = 6–9; Two-way ANOVA with Bonferroni’s post hoc test). G: Steady state glucose infusion rate was calculated by averaging GIRs from 80 to 120 min (n = 6–9; Student’s *t* test). H: Plasma glucose levels were determined by averaging the −10 and 0 min time points (n = 6–9; Student’s *t* test). I: The rate of glucose disappearance (Rd) was calculated for animals in the fasting state by averaging the Rd from −10 and 0 min time points and the clamped state by averaging Rd from 80 to 120 min (n = 6–9; Two-way ANOVA with Bonferroni’s post hoc test). J: Fold glucose disappearance was calculated by dividing clamp Rd by fasting Rd (n = 6–9; Student’s *t* test). Tissue specific uptake was measured in gonadal (K) and subcutaneous (L) white adipose tissue (n = 6–9; Student’s *t* test).

Obesity and T2D mellitus are commonly seen in subjects with NAFLD and it is well appreciated that excessive accumulation of lipids (i.e., “lipotoxicity) in the liver, skeletal muscle, and pancreatic beta cells can drive the overproduction of insulin and insulin resistance in target tissues (38, 39, 40). Recent studies have demonstrated that the hepatic expression of MBOAT7 is suppressed in obese humans and rodents (13, 14) and insulin treatment can acutely suppress MBOAT7 mRNA and protein expression (14). Furthermore, we demonstrated that ASO-mediated knockdown of *Mboat7* in HFD fed mice elicits profound hyperinsulinemia and systemic insulin resistance (13). However, several recent studies have demonstrated that genetic deletion of *Mboat7* specifically in hepatocytes (i.e., *Mboat7*^HSKO^ mice) does not alter insulin action or glucose tolerance (15, 17). To further understand the cell autonomous roles of *Mboat7* in insulin production and insulin action, we performed a series of studies comparing *Mboat7*^ASKO^ and *Mboat7*^HSKO^ mice (Fig. 3 and supplemental Fig. S2M–P). Despite having a severe fatty liver (supplemental Fig. S2I–K), *Mboat7*^HSKO^ mice have normal glucose tolerance versus *Mboat7*^flox/flox^ controls when challenged with a HFD (supplemental Fig. S2M). HFD fed *Mboat7*^HSKO^ mice also have similar levels of fasting blood glucose and leptin but modestly increased fasting insulin when compared to *Mboat7*^flox/flox^ controls (supplemental Fig. S2N–P). In contrast, when challenged with a HFD, *Mboat7*^ASKO^ mice exhibit impaired systemic glucose tolerance and increased fasting blood glucose and insulin levels (Fig. 3A–L). It is important to note that most metabolic studies were performed in male *Mboat7*^ASKO^ mice but many of the lipid changes and glucose intolerance phenotypes were also apparent in female *Mboat7*^ASKO^ mice (supplemental Fig. S4). When we subjected *Mboat7*^ASKO^ mice to an ITT to assess in vivo insulin sensitivity, there were some apparent differences between male and female mice (supplemental Fig. S5). When maintained on a chow diet, both male and female *Mboat7*^ASKO^ mice had similar ITT responses to those seen in *Mboat7*^flox/flox^ control mice (supplemental Fig. S5A–D). However, when challenged with a HFD, male *Mboat7*^ASKO^ mice had an unexpected increase in blood glucose levels, whereas HFD fed *Mboat7*^flox/flox^ control mice maintained glucose levels after an insulin challenge indicating clear HFD-induced insulin resistance (supplemental Fig. S5E, F). Whereas HFD fed female *Mboat7*^flox/flox^ control mice showed appreciable insulin-induced lowering of blood glucose, HFD fed *Mboat7*^ASKO^ mice showed significantly blunted insulin action (supplemental Fig. S5G, H). To further understand the underlying mechanism of insulin resistance, we performed euglycemic-hyperinsulinemic clamp studies in HFD fed control (*Mboat7*^flox/flox^) and *Mboat7*^ASKO^ mice (Fig. 3E–L and supplemental Fig. S5I–K). HFD fed *Mboat7*^ASKO^ mice exhibited reduced insulin sensitivity compared to HFD fed *Mboat7*^flox/flox^ mice as reflected by lower glucose infusion rate during the clamp (Fig. 3E–G). Importantly, the insulin resistance seen in HFD fed *Mboat7*^ASKO^ mice appears to be peripheral in nature given the blunted insulin-induced glucose disappearance (Fig. 3I, J) with no change to insulin-induced suppression of hepatic glucose production (supplemental Fig. S5I, J). Specifically, WAT insulin sensitivity is altered given gWAT specific uptake of 2-deoxyglucose (Rg) is reduced significantly and a similar trend is observed in scWAT (Fig. 3K, L). In contrast, there were no differences observed in glucose uptake in skeletal muscle, BAT, heart, or brain tissues (supplemental Fig. S5K). It is also interesting to note that gWAT tissue levels of 18:0- and 18:1-containing LPIs were significantly correlated with fasting blood glucose and glucose tolerance test area under the curve (supplemental Fig. S6). To look more broadly at whole body metabolism, we also subjected *Mboat7*^ASKO^ and *Mboat7*^ASKO^ mice fed HFD for 10 weeks to indirect calorimetry and found no differences in energy expenditure at thermoneutrality (30°C) or room temperature (23°C) (data not shown). Collectively, these data show for the first time that MBOAT7-driven LPI acylation in adipocytes, but not in hepatocytes, is critically important for the maintenance of circulating insulin and glucose levels as well as systemic insulin action.

### MBOAT7 is a critical metabolic regulator of adipose tissue lipid homeostasis and adipose tissue function

The proper storage of excess energy in the form of triacylglycerol (TAG) within adipose tissue is critically important for overall metabolic health. Although the major lipid class stored in WAT is TAG, adipocytes play a critically important role in regulating the levels of other lower abundance lipids that can shape local and systemic inflammatory and hormonal responses to influence T2D mellitus, NAFLD and other related metabolic diseases. To further understand how MBOAT7 impacts adipose tissue metabolism, we performed a series of comprehensive lipidomics studies in gWAT isolated from chow and HFD fed *Mboat7*^flox/flox^ control mice and *Mboat7*^ASKO^ mice. Although MBOAT7 has been previously shown to have exquisite substrate selectivity toward saturated LPIs and arachidonyl-CoA (41, 42, 43), we also analyzed the abundance of other free fatty acids, oxylipins, and diverse species of neutral lipids and glycerophospholipids. First, as a reference, it is notable that hepatocyte-specific deletion of *Mboat7* (*Mboat7*^HSKO^ mice) results in a large accumulation of substrate LPIs and a modest reduction in 38:4 PI in the liver but this is not apparent in gWAT (supplemental Fig. S2G–L). In contrast, *Mboat7*^ASKO^ mice do not show alteration in LPI or PI lipids in the liver (supplemental Fig. S1D–F) but do have elevations in 18:0- and 18:1-containing LPIs and large reductions in 38:4 PI in gWAT, especially in the context of HFD (Fig. 2B–D). *Mboat7*^ASKO^ mice also have reciprocal increases in more saturated PI species (34:2, 36:2, and 36:3) compared to *Mboat7*^flox/flox^ control mice in gWAT (Fig. 2D), but not liver (supplemental Fig. S1F). These results demonstrate that MBOAT7 plays a critical role in maintaining LPI and PI levels within the local tissue context and shows that MBOAT7 is the major enzymatic source of 38:4 in WAT (Fig. 2C) but a more minor contributor in the liver (supplemental Fig. S2H) (15, 16, 17).

Given the critical role that MBOAT7 plays in WAT LPI/PI homeostasis, we wanted to more comprehensively understand whether other lipid classes were altered in the WAT of *Mboat7*^ASKO^ mice. Other than modest alterations in 36:2 and 36:3 phosphatidylethanolamine (PE), adipocyte-specific deletion of *Mboat7* did not result in significant alterations of free fatty acids, non-LPI lysophospholipids, phosphatidylcholines, sphingomyelins, or ceramides (supplemental Fig. S7). Given the critical role that MBOAT7 plays in the Land’s cycle remodeling pathway (44), we also wanted to analyze arachidonic acid (AA)-derived oxylipins. When we quantified the WAT levels of >60 species of oxylipins, none were significantly different between *Mboat7*^flox/flox^ control mice and *Mboat7*^ASKO^ mice (supplemental Fig. S8). Consistent with this finding, we did not see any changes in *Pla2g4a* expression between *Mboat7*^flox/flox^ and *Mboat7*^ASKO^ mice (supplemental Fig. S9A). Also, when we quantified the levels of neutral lipids such as diacylglycerols and TAGs there were no significant differences between *Mboat7*^flox/flox^ control mice and *Mboat7*^ASKO^ mice when fed an HFD, except that select species of TAG (52:3, 56:4, 50:5, etc.) were modestly reduced in chow fed *Mboat7*^ASKO^ mice (supplemental Fig. S10). Despite the conservation seen in the major lipid classes found in WAT, *Mboat7*^ASKO^ mice did have some significant alterations in some complex lipids containing either AA (20:4, n-6) or eicosapentaenoic acid (EPA, 20:5, n-3) in WAT (supplemental Fig. S11) Specifically, some AA-containing PE species were increased in the WAT from *Mboat7*^ASKO^ mice (supplemental Fig. S11A); however, the expression of other AA-specific acyl transferase, *Lpcat3* (also known as *Mboat5*) was not changed (supplemental Fig. S9B). Furthermore, EPA-containing PI (38:5 PI) was dramatically reduced in *Mboat7*^ASKO^ mice, while other EPA-containing PS and PE species were slightly elevated (supplemental Fig. S11C). These data support the notion that adipocyte MBOAT7 plays a central role in WAT glycerophospholipid homeostasis locally but does not appreciably impact LPI and PI balance in the circulation or the liver.

**Fig. 4:**
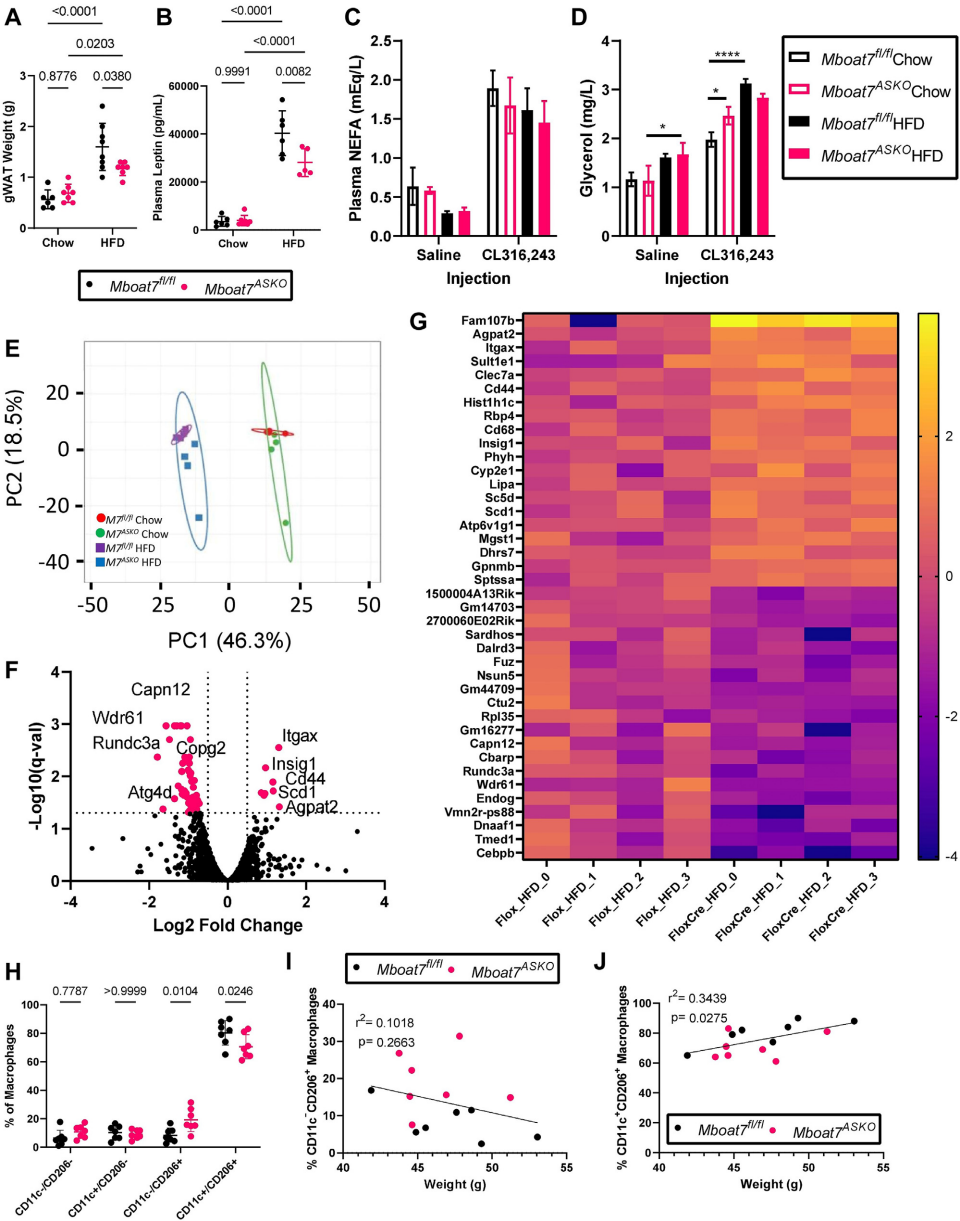
Adipocyte-Specific *Mboat7* Deletion (*Mboat7*^*ASKO*^) Reorganizes White Adipose Tissue Gene Expression and Circulating Adipokine Levels. Male control (*Mboat7*^*fl/fl*^) or adipocyte-specific Mboat7 knockout mice (*Mboat7*^*ASKO*^) were fed chow or high fat diet (HFD) for 20-week. A: gWAT weight measurements from *Mboat7*^*fl/fl*^ or *Mboat7*^*ASKO*^ mice fed Chow and HFD for 20 weeks (n = 6–8; Two-way ANOVA with Tukey’s *post hoc* test). B: Plasma Leptin was measured in *Mboat7*^*fl/fl*^ or *Mboat7*^*ASKO*^ mice that were fed a chow or HFD for 20 weeks (n = 5–7; Two-way ANOVA with Tukey’s *post hoc* test). To assess β3–adrenergic stimulated lipolysis, plasma nonesterified fatty acids (NEFA), (C) and glycerol (D) were measured in *Mboat7*^*fl/fl*^ or *Mboat7*^*ASKO*^ mice fed a chow or HFD for 11 weeks 15 min after saline or CL316,243 injection (n = 2–5/group; Three-way ANOVA with Tukey’s *post hoc* test). E–G: gWAT RNA was used for RNA-sequencing from *Mboat7*^*fl/fl*^ or *Mboat7*^*ASKO*^ mice that were fed a chow or HFD for 20 weeks. E: Groups clustered primarily based on the diet by principal component analysis (n = 4/group). F: A Volcano plot of transcripts was used to determine differentially expressed genes (DEGs) in *Mboat7*^*fl/fl*^ or *Mboat7*^*ASKO*^ mice that were fed an HFD for 20 weeks. Plot summarizes log2 fold changes versus significance in response to *Mboat7* inhibition (n = 4/group; genes with q-val < 0.05 and fold change > |0.5| were considered significantly differentially expressed). G: Row-normalized expression for the top 20 up and downregulated DEGs that reached a *P*-value <0.05 are shown by heat map in *Mboat7*^*fl/fl*^ or *Mboat7*^*ASKO*^ mice that were fed an HFD for 20 weeks. H: gWAT stromal vascular fraction was subjected to flow cytometry analysis of macrophage subpopulations. I, J: Correlation between gWAT macrophage subsets and body weight. All data are presented as mean ± S.D. unless otherwise noted.

Given the alterations in WAT lipid homeostasis in *Mboat7*^ASKO^ mice, we wanted to examine key aspects of adipose tissue function and cellularity within the adipose organ. Although body weight and total lean and fat mass were not significantly different between *Mboat7*^flox/flox^ control mice and *Mboat7*^ASKO^ mice after 8 weeks on diet (Fig. 2K–M), the weight of gWAT was significantly lower in *Mboat7*^ASKO^ mice after 20 weeks of HFD feeding (Fig. 4A), inverse of the significant increase in liver weight of *Mboat7*^ASKO^ animals at 20 weeks of HFD (Fig. 2F). However, less than 20 weeks of HFD feeding did not result in significant differences in liver or adipose tissue weights (supplemental Fig. S3A–D). Circulating levels of the adipocyte-derived hormone leptin were also reduced in HFD fed *Mboat7*^ASKO^ mice after 16 and 20 weeks of HFD (Fig. 4B and supplemental Fig. S3E). Next, we wanted to examine whether basal or catecholamine-stimulated lipolysis (i.e., a critical physiological function of WAT) was altered in*Mboat7*^ASKO^ mice. Although in general the circulating levels of lipolysis product glycerol and NEFA were not altered under basal conditions or stimulated conditions in *Mboat7*-deficient mice, chow fed *Mboat7*^ASKO^ mice did have elevations in circulating glycerol when treated with the β3-adrenergic receptor agonist CL-326,243 (Fig. 4C, D). Similarly, we did not observe significant changes in plasma glycerol, NEFA, or triglycerides in *Mboat7*^ASKO^ versus control mice under fasting or refeeding conditions (supplemental Fig. S3F–H). Next, we performed bulk RNA sequencing in gWAT. Under HFD feeding conditions, *Mboat7*^ASKO^ mice exhibited differential gene expression associated with altered lipid metabolism and immune cell populations (Fig. 4E–G). In particular, *Mboat7*^ASKO^ mice had elevated WAT mRNA expression of genes primarily expressed in macrophages including a cluster of differentiation 68 (*Cd68*), integrin alpha X (*Itgax*, encoding CD11c), C-type lectin domain containing 7a (*Clec7a*), among others (Fig. 4F, G) indicating the potential for an altered abundance of adipose tissue macrophages. To further understand whether the abundance of macrophages and other immune cell populations were altered in *Mboat7*^ASKO^ mice fed HFD for 16 weeks, we isolated the stromal vascular fraction from gWAT and performed flow cytometric analysis of immune cell populations (Fig. 4H–J and supplemental Fig. S12). Of the adipose tissue macrophage populations identified, *Mboat7*^ASKO^ mice had elevated levels of Cd11c-/Cd206+ cell populations and a reciprocal decrease in Cd11c+/Cd206+ double-positive cells (Fig. 4G). Although the relative percentage of Cd11c-/Cd206+ cells was not significantly correlated with body weight (Fig. 4H), the Cd11c+/Cd206+ double-positive cell population was significantly positively correlated with body weight in this cohort (Fig. 4I, J). In addition to alterations in macrophage subsets, HFD fed *Mboat7*^ASKO^ mice had elevated T cells and B cells compared to control (*Mboat7*^flox/flox^) mice (supplemental Fig. S12B–F). These data demonstrate that adipocyte MBOAT7-driven LPI acylation plays an important role in WAT lipid metabolic and immune cell homeostasis which can then shape systemic glucose tolerance.

### LPIs induce acute signaling events associated with insulin action in adipose tissue in a MBOAT7-dependent manner

**Fig. 5:**
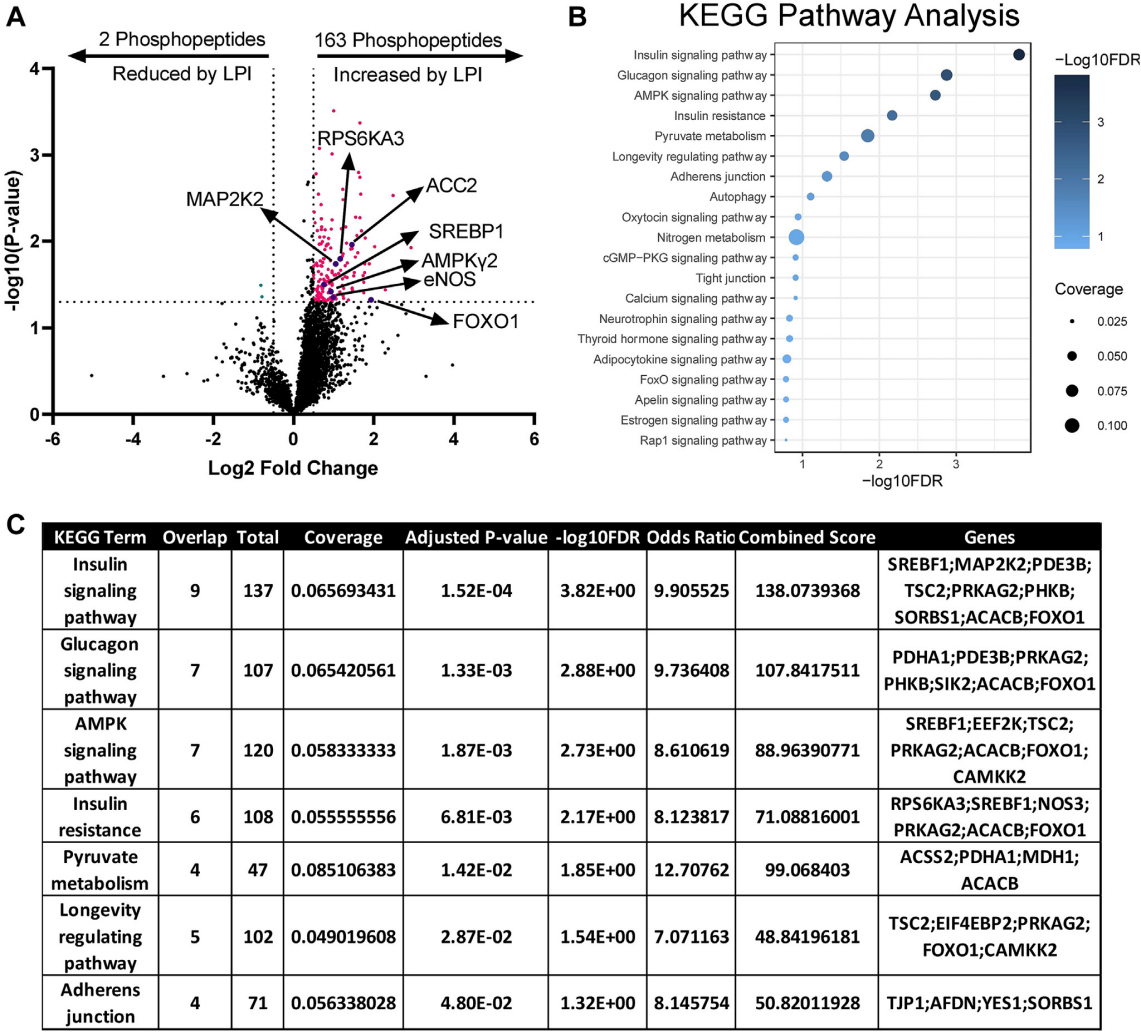
LPI-18:1 Induces Acute Signaling Events Associated with Insulin Action in Adipose Tissue of *Mboat7*^*ASKO*^ mice. A: Volcano plot of phosphopeptides upregulated and downregulated in gonadal white adipose tissue (gWAT) of *Mboat7*^*ASKO*^ mice. Phosphopeptides determined to be |log2FoldChange| >0.5 different in the LPI and saline samples with a *P*-value <0.05 (two-tailed *t* test) were considered significantly differentially phosphorylated. (Average of n = 4/group; pink dots represent significantly upregulated phospho-peptides, green dots represent significantly downregulated phospho-peptides; and purple dots represent insulin resistance-associated phosphopeptides that are significantly different). B, C: KEGG pathway analysis of significantly differentially phosphorylated peptides. KEGG, Kyoto Encyclopedia of Genes and Genomes; LPI, lysophosphatidylinositol; *Mboat7*^ASKO^, *Mboat7* adipocyte-specific knockout mice.

Given that *1*) LPIs accumulate in gWAT of HFD fed and *Mboat7*^*ASKO*^ mice (Fig. 2B and supplemental Fig. S4A), *2*) *Mboat7*^*ASKO*^ results in profound glucose intolerance and insulin resistance (Fig. 3, supplemental Figs. S4H, I and S5), and *3*) LPIs correlate with fasting blood glucose and area under the curve during glucose tolerance testing (supplemental Fig. S6B–D), we hypothesized that LPIs may mediate signaling associated with insulin resistance. To test this hypothesis, we injected chow fed *Mboat7*^*flox/flox*^ and *Mboat7*^*ASKO*^ mice with saline or LPI-18:1 and collected gWAT acutely after 15 min. To unbiasedly identify LPI-driven signaling events we performed global phosphoproteomic analysis of isolated gWAT and found that. *Mboat7*^*ASKO*^ mice upregulate 163 phosphopeptides in response to LPI but not in control *Mboat7*^*flox/flox*^ mice. Using KEGG pathway analysis, we find that indeed LPI-18:1 significantly induces a phosphopeptide signature associated with insulin signaling and insulin resistance in mice lacking MBOAT7 function in adipocytes (*Mboat7*^*ASKO*^ mice) (Fig. 5A–C). In contrast, mice with intact MBOAT7 function (*Mboat7*^*flox/flox*^ mice) show only 40 significantly altered phosphopeptides and no significantly altered KEGG pathways when challenged with exogenous LPI (supplemental Fig. S13). Together, these data indicate that LPI-18:1 can alter the gonadal adipose tissue phosphoproteome in a MBOAT7-dependent manner and this is associated with pathways linked to insulin resistance.

## Discussion

Since the original genome wide association studies study by Buch *et al.* (8) linking the rs641738 SNP near *MBOAT7* to liver disease, there has been rapid progress in our understanding of how *MBOAT7* is mechanistically linked to the progression of alcohol-associated liver disease, NAFLD, and viral-driven liver injury. The clear association between MBOAT7 loss-of-function and diverse liver diseases serves as yet another example of how genetics can powerfully identify new pathways relevant to human disease. It also supports the long-standing notion that abnormal lipid metabolism initiates liver injury. Since 2019, several animal studies have likewise demonstrated that *Mboat7* loss-of-function in mice is sufficient to drive NAFLD progression (13, 14, 15, 16, 17). This article builds on our initial observation that ASO-mediated knockdown of *Mboat7* promotes NAFLD progression, hyperinsulinemia, and insulin resistance in mice (13). Here we have further clarified the cell autonomous roles of *Mboat7* in HFD-driven metabolic disturbance by comparing metabolic phenotypes in *Mboat7*^ASKO^ and *Mboat7*^HSKO^ mice. The major findings of the current study include the following: *1*) *Mboat7* expression in WAT is negatively correlated with adiposity across the strains represented in the HMDP, *2*) Hepatocyte-specific deletion of *Mboat7* (*Mboat7*^HSKO^) promotes fatty liver and liver injury but does not alter tissue insulin sensitivity in HFD fed mice, *3*) Adipocyte-specific deletion of *Mboat7* (*Mboat7*^ASKO^) under chronic HFD feeding promotes mild fatty liver, clear hyperinsulinemia, and systemic insulin resistance in HFD fed mice, *4*) MBOAT7 is the major source of AA containing PI (38:4 PI) and also indirectly impacts other glycerophospholipids in gWAT, *5*) MBOAT7 function in gWAT does not alter the tissue abundance of AA-derived oxylipins, *6*) MBOAT7 function in gWAT impacts circulating leptin levels, and the immune cell landscape in WAT under HFD feeding, and *7*) acylation of LPIs by MBOAT7 in WAT limits proinsulin resistance signaling. Collectively, these data support a clear cell autonomous role for MBOAT7-driven acylation of LPI lipids as a key protective mechanism against obesity-linked NAFLD progression, hyperinsulinemia, and systemic insulin resistance. These data strongly suggest that MBOAT7 is an important contributor to multiple aspects of the metabolic syndrome, which is regulated by the combination of MBOAT7 function in hepatocytes, adipocytes, and likely other cell types that contribute to tissue inflammation and fibrosis.

One of the key findings of this work is that MBOAT7 function in adipocytes is critically important for the maintenance of euglycemia in obese mice (Fig. 3). In contrast, genetic deletion of *Mboat7* in hepatocytes is sufficient to drive hepatic steatosis but does not drastically alter hyperinsulinemia or insulin sensitivity in either chow or HFD fed mice (supplemental Fig. S2). Interestingly, our studies show drastic effects on systemic glucose tolerance and insulin resistance in HFD fed *Mboat7*^ASKO^ mice yet our euglycemic-hyperinsulinemic studies indicate no change in suppression of hepatic glucose production by insulin and only modest decreases in insulin-stimulated glucose uptake by white adipose depots (Fig. 3 and supplemental Fig. S5). Because these depots show less glucose uptake activity than other tissues that account for the majority of glucose disposal, such as skeletal and cardiac muscle, BAT, and the brain, there is likely other mechanisms that contribute to these systemic effects. While glucose intolerance and insulin resistance was obvious in HFD fed *Mboat7*^ASKO^ mice, these phenotypes are not significant in chow fed *Mboat7*^ASKO^ mice, which may be explained in part by significant increases in β3-adrenergic activation (Fig. 4D), which has been previously shown to enhance fatty acid oxidation in adipose tissue of mice and humans (45, 46).

In addition to these findings regarding local WAT defects, these studies raise the question of how MBOAT7 in adipocytes controls insulin secretion in the pancreas and fat accumulation in the liver. The most straightforward explanation is that alterations in either the substrates (LPIs or fatty acyl-CoAs) or products (38:4 PI) of the MBOAT7 reaction initiate endocrine lipid signaling effects. We originally hypothesized that when adipocyte MBOAT7 function is lost, substrate LPIs would accumulate in both the WAT as well as the circulation to initiate a WAT-to-pancreas endocrine axis to stimulate insulin overproduction. In support of this concept, several published reports show that LPI lipids can stimulate glucose-stimulated insulin secretion in pancreatic beta cells in culture (47, 48). Furthermore, 18:1 LPI has also been shown to stimulate the key metabolic hormone glucagon-like peptide in intestinal enteroendocrine *L*-cells to further shape insulin secretion (49, 50). However, our results suggest that although WAT tissue of LPI is dramatically increased in *Mboat7*^ASKO^ mice (Fig. 2B), circulating levels of LPI lipids are unaltered in *Mboat7*^ASKO^ mice (supplemental Fig. S1). These data suggest this potential lipid-mediated endocrine axis is unlikely, although there is a clear LPI-mediated autocrine or paracrine axis within WAT (Fig. 5). It is important to note however that LPI lipids can exhibit altered signaling potential when bound to albumin versus being carried on plasma lipoproteins (51), so additional work is required to determine whether adipocyte MBOAT7 activity selectively impacts albumin-conjugated versus lipoprotein-associated LPI levels. In addition to an LPI-endocrine signaling axis, it is possible that MBOAT7 can impact adipose tissue homeostasis and systemic insulin sensitivity via other mechanisms. It remains possible that MBOAT7 product PI lipids including 38:4 PI and resulting phosphoinositides may also play a key role in the phenotypes observed. Another possibility to consider is that glycerophosphatidylinositol-linked proteins may be altered in *Mboat7*^ASKO^ mice given that PIs are required for glycerophosphatidylinositol synthesis (52). These possibilities deserve further exploration. Another interesting observation coming from the current study is the different molecular weights of MBOAT7 observed in crude tissue lysates (Fig. 2A) versus the consistent size in microsomal fractions (supplemental Fig. S2A), which could suggest that multiple MBOAT7 isoforms exist in different tissues and may localize to organelles other than the endoplasmic reticulum. In conclusion, this work shows that MBOAT7 function in adipocytes and hepatocytes play unique roles in shaping HFD-driven metabolic disturbance and further supports the notion that the LPI-MBOAT-PI axis may have untapped therapeutic potential in obesity-related insulin resistance and NAFLD progression.

## Data availability

RNA sequencing data can be accessed at GEO Profiles at the NCBI: GSE203414, the mass spectrometry phospoproteomics data have been deposited to the ProteomeXchange Consortium via the PRIDE (34) partner repository with the dataset identifier PXD039894 and 10.6019/PXD039894 and full accession will be available upon acceptance of this work. All materials, methods, and datasets included in this article is readily available upon request.

## Supplemental data

This article contains supplemental data (53, 54).

## Conflict of interest

Dr. Daniela Allende reports serving as an Advisory Board Member for Incyte Corporation. All other authors declare no competing financial interests related to this work.
