# Supplementary material for: MBOAT7-driven lysophosphatidylinositol acylation in adipocytes contributes to systemic glucose homeostasis
Source: J Lipid Res. 2023 Feb 18;64(4):100349. doi: 10.1016/j.jlr.2023.100349 (PMC10041558; doi:10.1016/j.jlr.2023.100349)
Supplement: Supplemental Figures S1–S13 and Table S1 [file mmc3.docx]

**Supplemental Material for MBOAT7-Driven Lysophosphatidylinositol Acylation in Adipocytes Contributes to Systemic Glucose Homeostasis.**

**Methods**

**Untargeted lipidomics and oxylipin quantification**

Lipidomics and oxylipin data were acquired at the UC Davis West Coast Metabolomics Center. For complex lipid analyses, reverse phased liquid chromatography-tandem mass spectrometry (RPLC-MS/MS) was used to perform lipidomic analysis and began by adding 100 μL run solvent (9
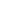
:
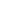
1 methanol/toluene (v/v)) to microcentrifuge tubes from the dried upper layer of extraction. Chromatography was performed using an Agilent 1290 UHPLC and mass spectra were collected with an Agilent 6550 QTOF mass spectrometer. An Acquity UPLC CSH C18 (100 mm × 2.1 mm, 1.7 μm particle size) column (Waters, Milford, MA) with an Acquity UPLC CSH C18 (5 mm × 1.2 mm, 1.7 μm particle size) pre-column (Waters, Milford, MA) was used with mobile phase A (6
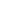
:
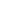
4 acetonitrile/water (v/v)) and mobile phase B (9
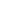
:
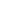
1 isopropanol acetonitrile (v/v)). Mobile phases A and B were modified with 10 mM ammonium formate and 0.1% formic acid for positive mode ionization and 10 mM ammonium acetate for negative mode ionization. The LC gradient started at 15% B, increased to 30% B from 0–2 minutes, increased to 48% B from 2–2.5 minutes, increased to 82% B from 2.5–11 minutes, increased to 99% B from 11–11.5 minutes, held at 99% B from 11.5–12 minutes, returned to 15% B from 12–12.1 minutes and held at 15% B from 12.1–15 minutes. The autosampler was held at 4 °C and needle wash was performed before and after sample injections for 10 seconds with isopropanol. Injection volumes were 4 μL for both positive and negative mode ionization analyses. Spectral data was collected with a scan range of 120–1700 m/z. MS/MS fragmentation used data-dependent-acquisition (DDA) and was collected for the top 4 most abundant ions from each MS scan. LC-MS/MS data were processed using open source software MS-DIAL (43) (version 4.24) which performed peak picking, deisotoping, automated peak annotation, alignment and gap filling.  Blank subtraction was performed by removing features that had a maximum sample intensity/average blank intensity ratio of less than 5. Adduct and duplicate features were flagged using Mass Spectral Feature List Optimizer (MS-FLO) (43). Data from each of the four analytical platforms (RPLC-MS/MS ESI+/−, HILIC-MS/MS ESI+/−) were processed separately and combined after data curation. No data normalization was performed because no trend in data intensities was observed from the internal standards during data acquisition. Peak height was used for all quantitation. Metabolite annotations were made using defined confidence levels (43) based on accurate mass, MS/MS library matching to experimental data, and retention time from authentic standards run on the same instrument.  Tandem MS/MS libraries of the MassBank of North America ([MassBank.us](https://protect-us.mimecast.com/s/dVSbCZ6yPEtMlLPOzsyFPla?domain=massbank.us)) and NIST17 (NIST, Gaithersburg, MD) were used for spectral matching. A manual curation of datasets was performed to reduce in-source fragment annotations identified by very similar RT and high correlation between features. Manual review of MS/MS matches was performed to remove poor spectral matches since false positive annotations can occur when automatically matching MS/MS from complex biological samples to large MS/MS spectral libraries (43). For oxylipin analyses, samples were added to a 96-well plate followed sequentially by 25 μL anti-oxidant solution, 25 μL of surrogate standards in methanol, 25 μL of CUDA and PHAU standards in methanol, and 125 μL acetonitrile/methanol 1:1. Plates were then vortexed for 30 s, centrifuged at 6 °C for 5 min at 15,000 rcf and filtered through a PVDC 0.2-micron filter plate. Plates were then sealed and kept at −20 °C until analysis. Extracted oxylipins were separated and quantified using a Waters i-Class Acquity UHPLC system coupled to a Sciex 6500+ QTRAP mass spectrometer operated in negative ionization mode. Oxylipins were quantified by targeted, retention time-specific, multiple reaction monitoring ion transitions. A total of 78 oxylipins were targeted, and targets that appeared in any sample above the signal-to-noise ratio of 3:1 were quantified. This resulted in quantified values for 27 n-6-related oxylipins, including 17 ARA-related and 10 LA-related oxylipins. There were 12 n-3-related oxylipins measured, which included 6 ALA-related, 4 DHA-related, and 2 EPA-related oxylipins. Oxylipins measured in a sample below the limit of quantitation (LOQ), defined as signal-to-noise ratio below 3:1, were converted to 10% of the LOQ.

**Quantitative Real-Time Polymerase Chain Reaction (qRT-PCR)**

qRT-PCR analysis was conducted as previously described (2). cDNA was prepared using a qScript supermix (QuantaBio, Beverly MA, USA, 95048). qRT-PCR was carried out using SYBR Fast reagents (Applied Biosystems, Waltham, MA, USA, 4385618) on an Applied Biosystems StepOne Plus machine. Data were analyzed using the ΔΔCT method, and expression was normalized such that the chow fed *Mboat7*^flox/flox^ group’s expression equaled 1. All primer sequences are listed in the Key reagents table (**Supplemental Table 1**).

**References**

1. Folz JS, Shalon D, Fiehn O. 2021. Metabolomics analysis of time-series human small intestine lumen samples collected in vivo**.** *Food & Function* **12:**9405-9415.

2. Massey W, Osborn LJ, Banerjee R, Horak A, Fung KK, Orabi D, Chan ER, Sangwan N, Wang Z, Brown JM. Flavin-Containing Monooxygenase 3 (FMO3) Is Critical for Dioxin-Induced Reorganization of the Gut Microbiome and Host Insulin Sensitivity. Metabolites. 2022 Apr 18;12(4). doi: 10.3390/metabo12040364. PubMed PMID: 35448550; PubMed Central PMCID: PMC9029240.

**Supplemental Figures and Legends**

**
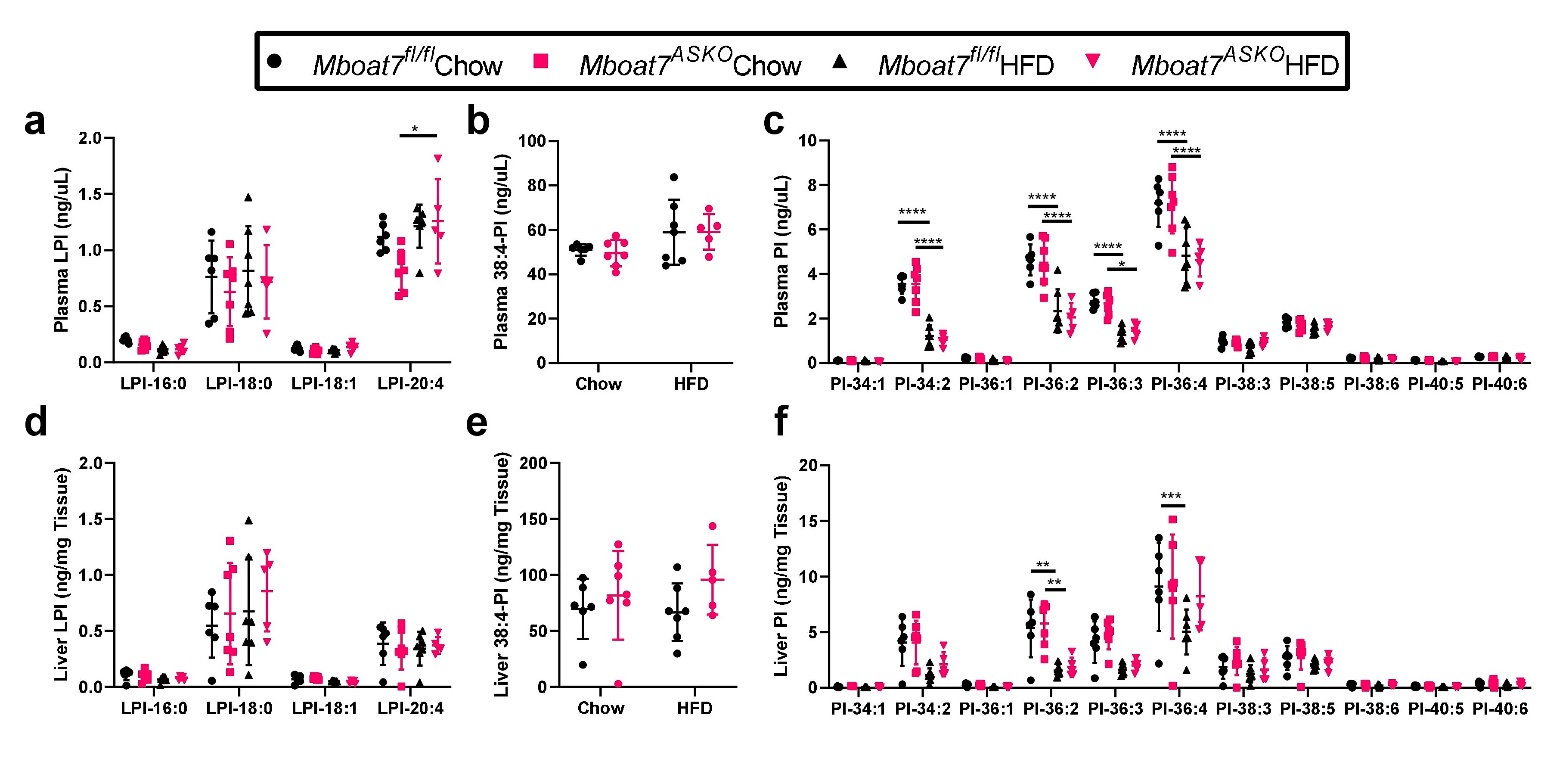
**

**Supplemental Fig. 1. Plasma and Liver LPI and PI Levels are Not Altered in *Mboat7^ASKO^* Mice.** Male control (*Mboat7^fl/fl^*) or adipocyte-specific Mboat7 knockout mice (*Mboat7^ASKO^*) were fed chow or high fat diet (HFD) for 20-weeks. Plasma LPI (**a**), PI-38:4 (**b**), and other PI species (**c**) were, were quantified via LC-MS in *Mboat7^fl/fl^* or *Mboat7^ASKO^* mice (n=5-7/group; *****P*≤0.0001; Two-way (**b**) or Three-way (**a,c**) ANOVA with Tukey’s *post-hoc* test). Liver LPI (**d**), PI-38:4 (**e**), and other PI species (**f**) were, were quantified via LC-MS in *Mboat7^fl/fl^* or *Mboat7^ASKO^* mice were fed chow or HFD for 20-weeks (n=5-7/group; *****P*≤0.0001; Two-way (**e**) or Three-way (**d,f**) ANOVA with Tukey’s *post-hoc* test). All data are presented as mean ± S.D.

**
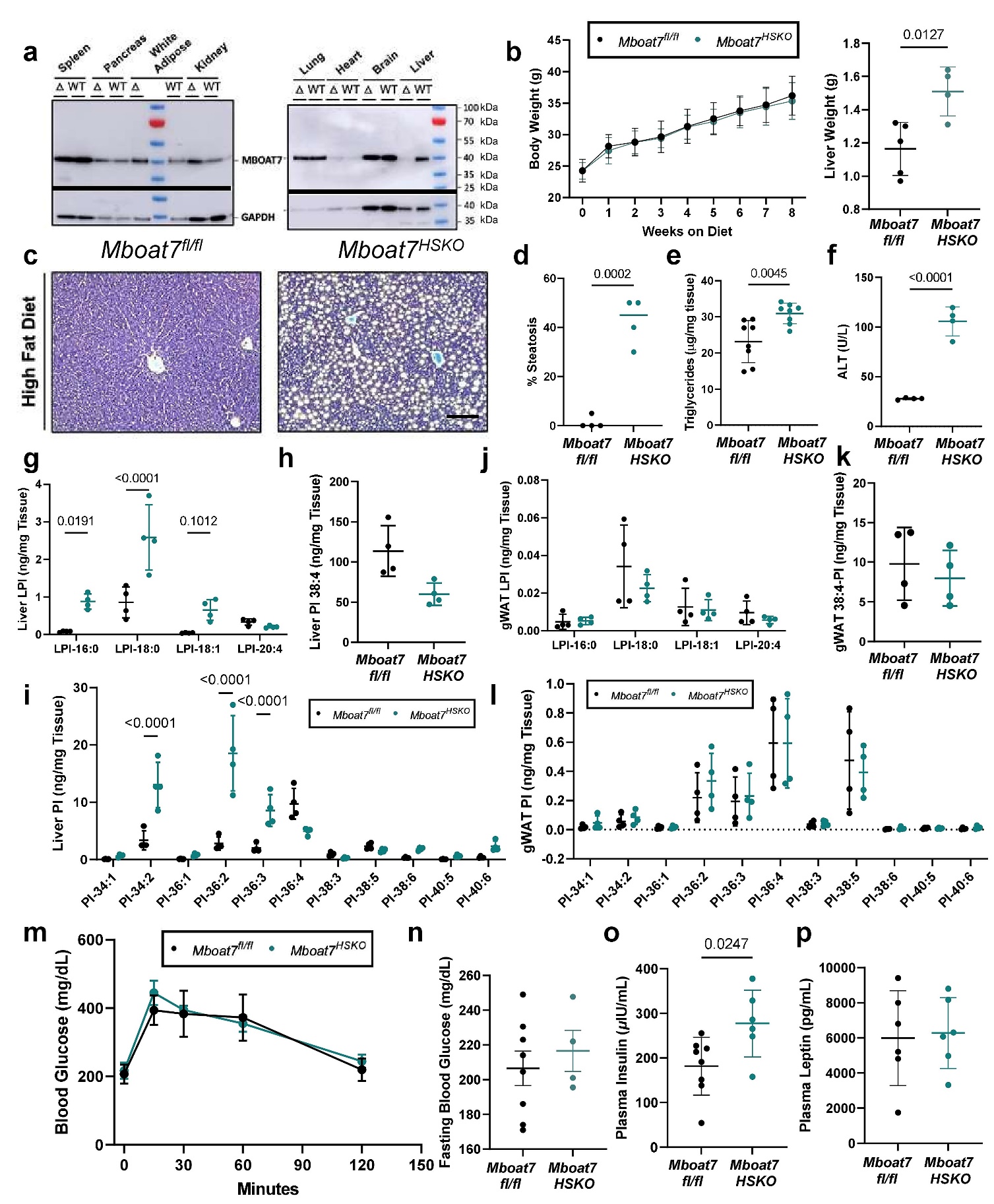
**

**Supplemental Fig. 2. High Fat Diet Feeding in Hepatocyte-Specific *Mboat7* Knockout Mice (*Mboat7^HSKO^*) Results in Profound Fatty Liver Without Altering Glucose Tolerance.** Male control (*Mboat7^fl/fl^*) or hepatocyte-specific Mboat7 knockout mice (*Mboat7^HSKO^*) were fed chow or high fat diet (HFD) for 8-weeks and metabolically phenotyped. (**a**) Western blots on microsomal fractions from hepatocyte-specific *Mboat7* deletion (*Mboat7^HSKO^*) tissues were probed for MBOAT7 and GAPDH to show a specific reduction of MBOAT7 protein in the liver. (**b-l**) *Mboat7^fl/fl^* or *Mboat7^HSKO^* fed a HFD for 10 weeks. (**b**) Body weight curve (n=6-8/group; Two-way ANOVA with Tukey’s *post-hoc* test). (**c**) Representative liver hematoxylin and eosin stained sections. 20x magnification (scale bar=400µm). (**d**) Percent steatosis was quantified by a blinded pathologist (n=4/group; Two-sided Student’s t-test). Hepatic triglycerides (**e**) and plasma alanine aminotransferase (ALT) (**f**) were measured enzymatically (n=8/group) (**e**) or 4 (**f**); Two-sided Student’s t-test). Liver LPI (**g**), PI-38:4 (**h**), and other PI species (**i**) were, were quantified via LC-MS in *Mboat7^fl/fl^* or *Mboat7^HSKO^* mice were fed HFD for 10-weeks (n=4/group; Two-sided Student’s t-test (**h**) or Two-way ANOVA with Tukey’s *post-hoc* test (**g,i**)). gWAT LPI (**j**), PI-38:4 (**k**), and other PI species (**l**) were, were quantified via LC-MS in *Mboat7^fl/fl^* or *Mboat7^HSKO^* mice were fed chow or HFD for 10-weeks (n=5-7/group; *****P*≤0.0001; Two-sided Student’s t-Test (**k**) or Two-way ANOVA with Tukey’s *post-hoc* test (**j,l**). (**m-n**) *Mboat7^fl/fl^* or *Mboat7^HSKO^* mice were fed an HFD for 3-4 weeks and then underwent an intraperitoneal glucose tolerance test (GTT). (**m**) Plasma glucose levels were measured (in duplicate or triplicate at each time point) throughout the GTT (n=4-8; Two-way ANOVA with Tukey’s *post-hoc* test). (**n**) Fasting blood glucose was measured after a four hour fast (time=0 minutes for GTT) (n=4-8/group; Two-sided Student’s t-Test). Fasting plasma insulin (**o**) and leptin (**p**) was measured in *Mboat7^fl/fl^* or *Mboat7^HSKO^* fed an HFD for 10 weeks. Data in (**a-l,o,p**) are presented as mean ± S.D. Data in (**m,n**) are presented as mean ± S.E. M. (blood glucose readings during GTT were taken in duplicate or triplicate if duplicate measures varied by >10%).

**
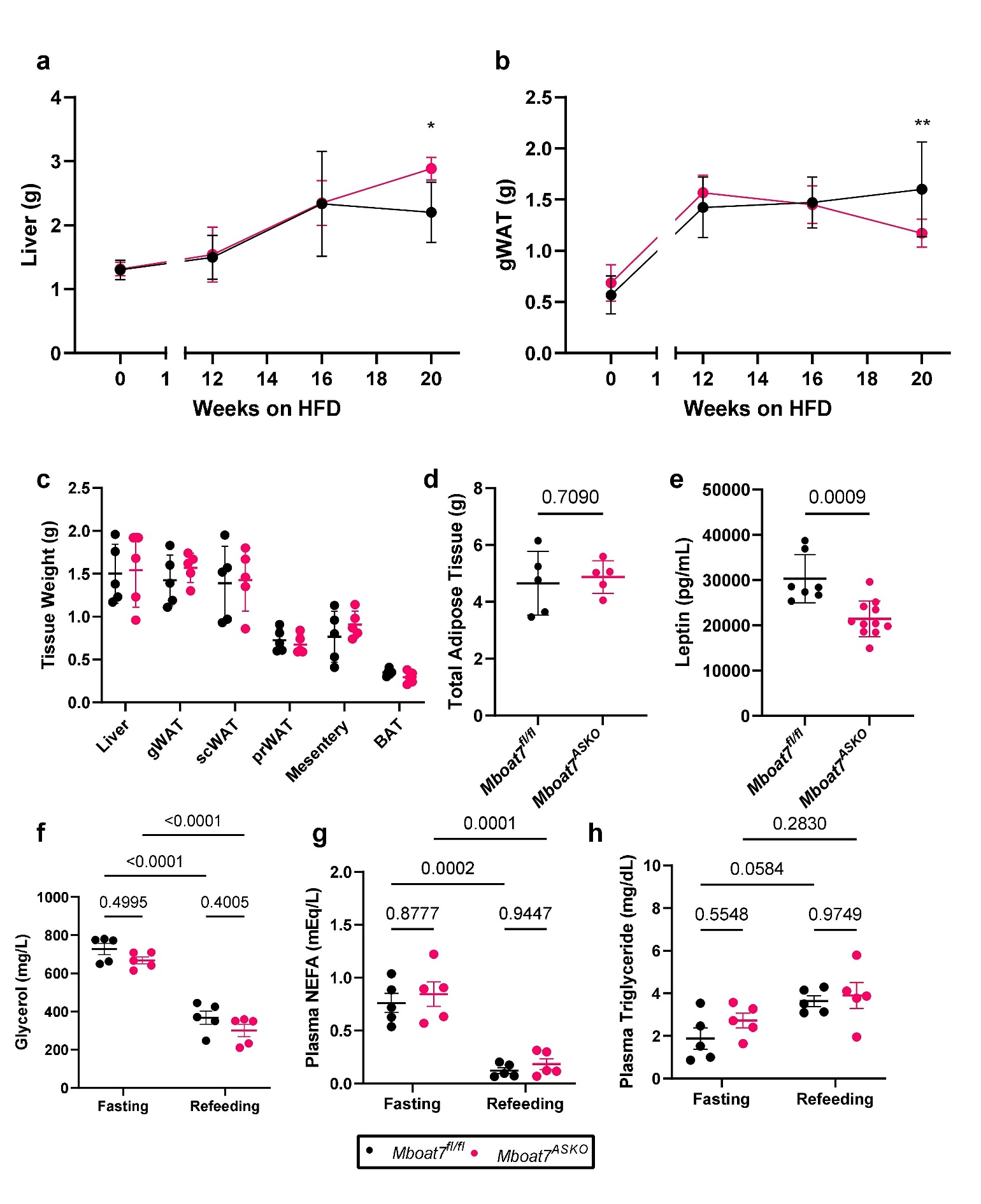
**

**Supplemental Fig. 3. *Mboat7^ASKO^* Display phenotypic alterations only after long-term HFD feeding.** Liver (**a**) and gWAT (**b**) are only significantly different after 20 weeks of HFD feeding. Data are from 4 independent experiments; are presented as mean ± S.D. 0 weeks of HFD feeding mice are represented by chow fed arm from 20 week study. (n=5-11/group; **P*≤0.05, ***P*≤0.01; Two-way way ANOVA with Bonferroni’s *post-hoc* test). (**c,d,f-h**) In 12 week HFD feeding study, mice were fasted overnight (1600-0800, 16 hours) and then refed with HFD for 3 hours before necropsy. Individual tissue weights (**c**), total adipose tissue weight (**d**), and plasma lipid fasting/refeeding responses (**f-h**) are unchanged. Data are presented as mean ± S.D. (n=5/group; **P*≤0.05, ***P*≤0.01; Two-way way ANOVA with Bonferroni’s *post-hoc* test (**c**) or Tukey’s *post-hoc* test (**f-h**) or Two-sided Student’s t-Test) (**d**)). Consistent with 20 weeks of HFD feeding, plasma leptin (**e**) was significantly reduced in *Mboat7^ASKO^* mice following 16 weeks of HFD feeding. Data are presented as mean ± S.D. (n=7-11/group; Two-sided Student’s t-Test)).

**
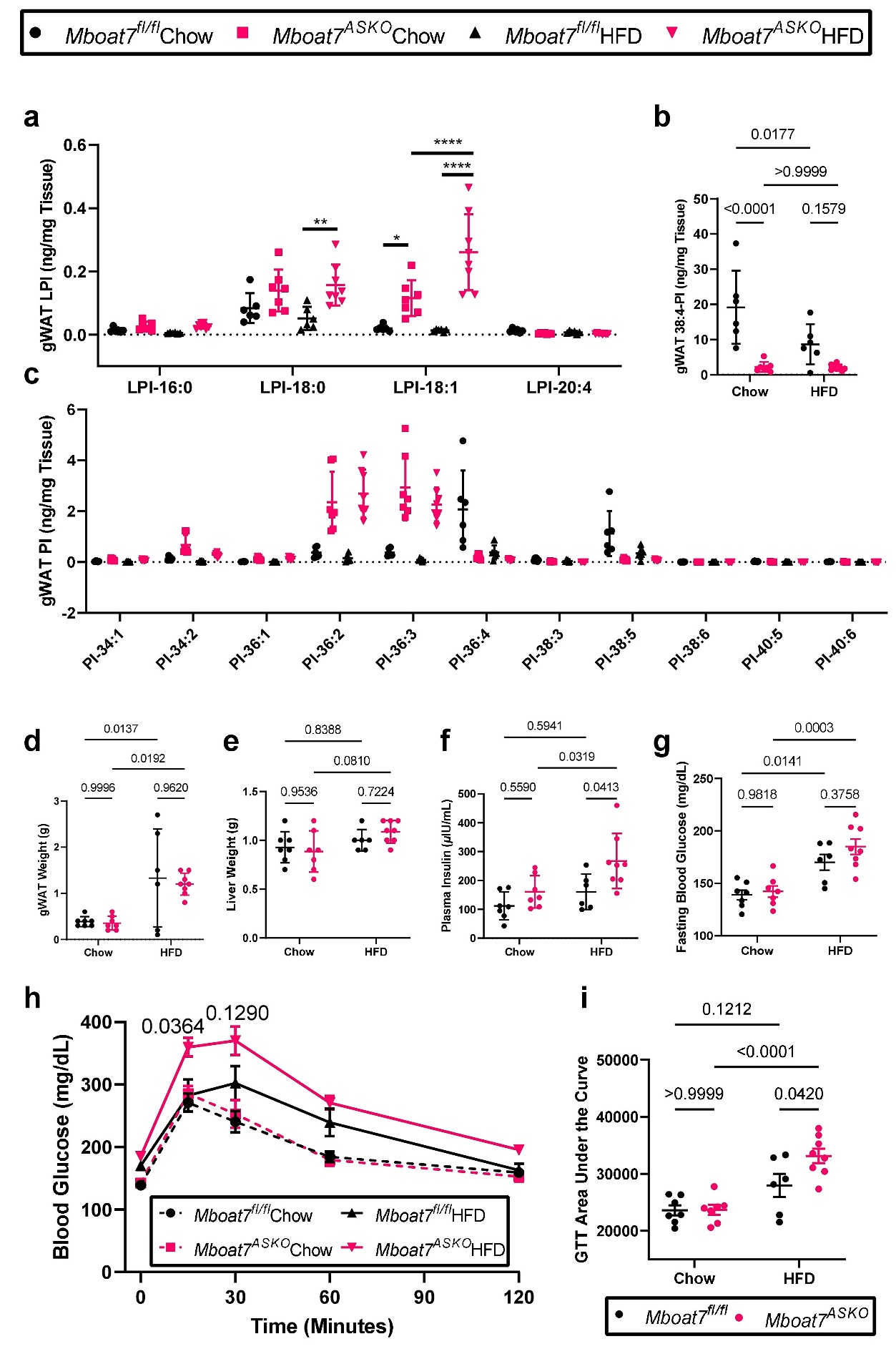
**

**Supplemental Fig 4. Female *Mboat7^ASKO^* Display Altered LPI/PI Balance in Adipose Tissue and Have Impaired Glucose Tolerance.** Gonadal white adipose tissue **(**gWAT) Lysophosphatidylinositol (LPI) (**a**) and phosphatidylinositol (PI) species, including the MBOAT7 product PI-38:4 (**b**) and others (**c**), were quantified via LC-MS in *Mboat7^fl/fl^* or *Mboat7^ASKO^* mice were fed chow or HFD for 20-weeks (n=5-7/group; *****P*≤0.0001; Two-way (**b**) or Three-way (**a,c**) ANOVA with Tukey’s *post-hoc* test). gWAT (**d**) and liver (**e**) weight measurements from female *Mboat7^fl/fl^* or *Mboat7^ASKO^* mice fed Chow and HFD for 20 weeks (n=6-8/group; Two-way ANOVA with Tukey’s *post-hoc* test). (**f**) Fasting plasma insulin was measured in *Mboat7^fl/fl^* or *Mboat7^ASKO^* mice that were fed a chow or HFD for 20 weeks (n=6-8/group; Two-way ANOVA with Tukey’s *post-hoc* test). (**g-i**) Female *Mboat7^fl/fl^* or *Mboat7^ASKO^* mice were fed a chow or HFD for 12 weeks and then underwent an intraperitoneal glucose tolerance test (GTT). (**g**) Fasting blood glucose was measured after a four hour fast (time=0 minutes for GTT) (n=6-8/group; Two-way ANOVA with Tukey’s *post-hoc* test). (**h**) Plasma glucose levels were measured (in duplicate or triplicate at each timepoint) throughout the GTT (n=6-8/group; Three-way ANOVA with Tukey’s *post-hoc* test). (**i**) Area under the curve was calculated for each mouse throughout the GTT (n=6-8/group; Two-way ANOVA with Tukey’s *post-hoc* test). Data in (**a-f**) are presented as mean ± S.D. Data in (**g-i**) are presented as mean ± S.E. M. (blood glucose readings during GTT were taken in duplicate or triplicate if duplicate measures varied by >10%).

**
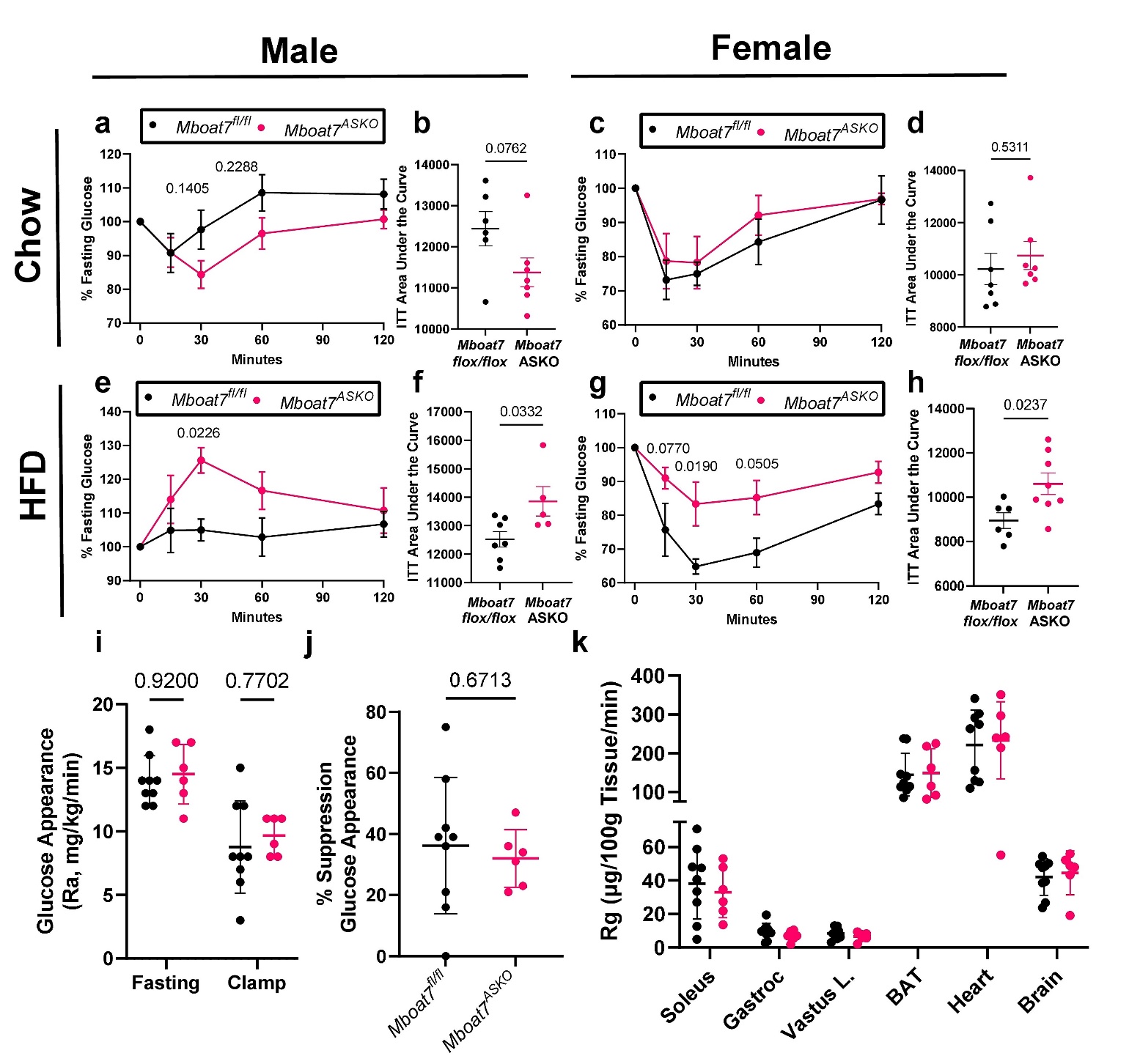
**

**Supplemental Fig. 5. Adipocyte-Specific *Mboat7* Deletion (*Mboat7^ASKO^*) Promotes Peripheral, but not Central, Insulin Resistance.** (**a-c**) Male or female control (*Mboat7^fl/fl^*) or adipocyte-specific Mboat7 knockout mice (*Mboat7^ASKO^*) were fed a chow or HFD for 14 weeks and then underwent an insulin tolerance test (ITT). Plasma glucose levels were measured (in duplicate or triplicate at each timepoint) throughout the ITT and area under the curve (AUC) was calculated (n=5-7; Three-way ANOVA with Tukey’s *post-hoc* test). (**i-k**) Male control (*Mboat7^fl/fl^*) or adipocyte-specific Mboat7 knockout mice (*Mboat7^ASKO^*) were fed HFD for 12-13 weeks, underwent surgery for catheterization of carotid artery and a jugular vein, and were subjected to euglycemic-hyperinsulinemic clamping. (**i**) The rate of glucose disappearance (Rd) was calculated for animals in the fasting state by averaging the Rd from -10 and 0 minute time points and the clamped state by averaging Rd from 80-120 minutes (n=6-9/group; Two-way ANOVA with Bonferroni’s *post-hoc* test). (**j**) Fold glucose disappearance was calculated by dividing clamp Rd by fasting Rd (n=6-9/group; Two-sided Student’s t-Test)). (**k**) Tissue specific uptake was measured in skeletal muscles (gastroc, soleus, vastus), brown adipose tissue (BAT), heart, and brain (n=6-9/group; Two-sided Student’s t-Test).

**
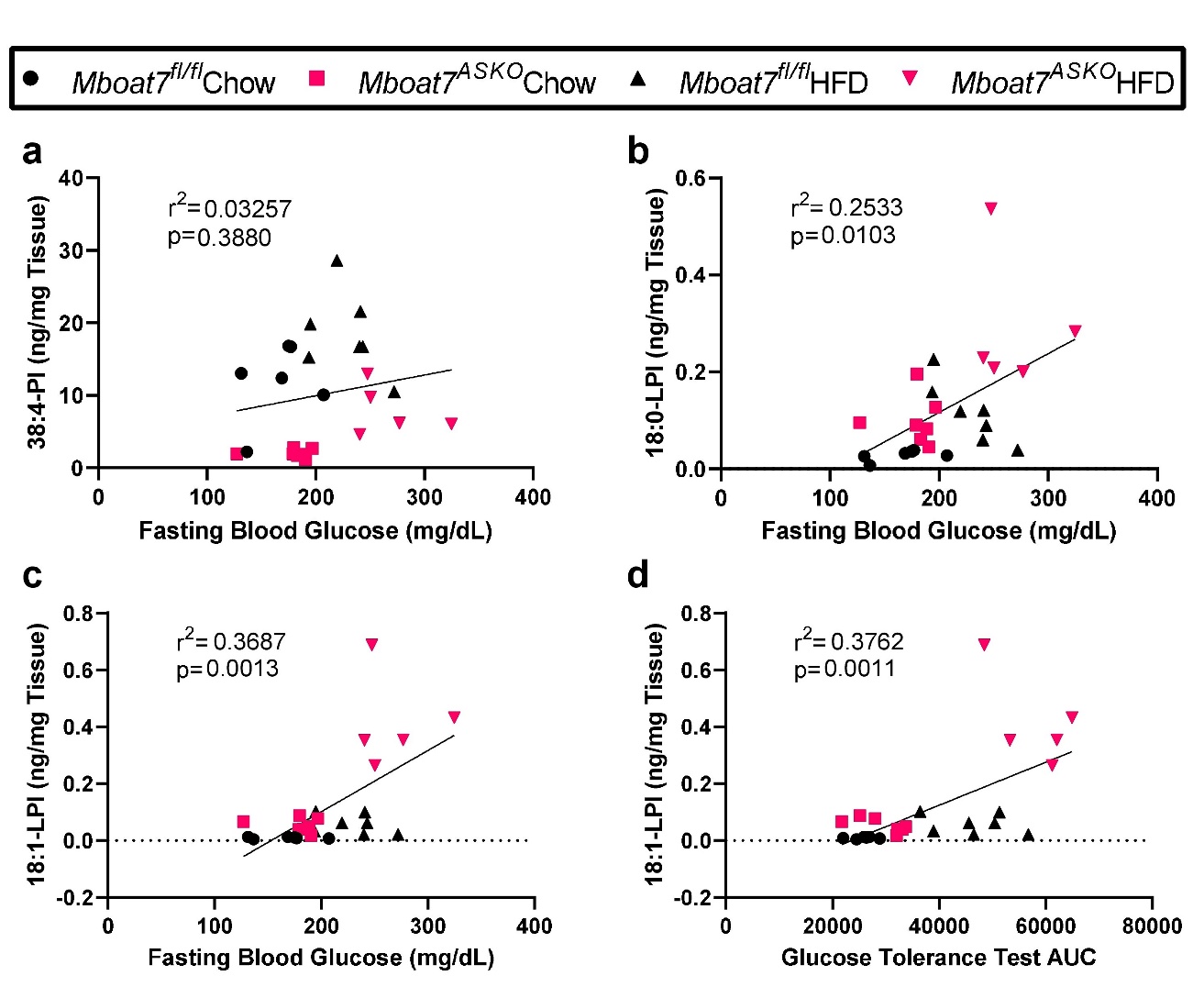
**

**Supplemental Fig. 6. Adipose Tissue LPI Levels Correlate with Systemic Glucose Tolerance.** Male control (*Mboat7^fl/fl^*) or adipocyte-specific Mboat7 knockout mice (*Mboat7^ASKO^*) were fed chow or high fat diet (HFD) for 20-weeks and correlation analysis was performed for glucose phenotypes and MBOAT7 substrate lysophosphatidylinositols (LPI) and product phosphatidylinositol (PI) lipids. (**a**) gWAT PI-38:4 at end of the experiment vs Fasting Blood Glucose at GTT. gWAT LPI-18:0 (**b**) and LPI-18:1 (**c**) at end of the experiment vs Fasting Blood Glucose at GTT. (**d**) gWAT LPI-18:1 at end of the experiment vs GTT Area under the curve.

**
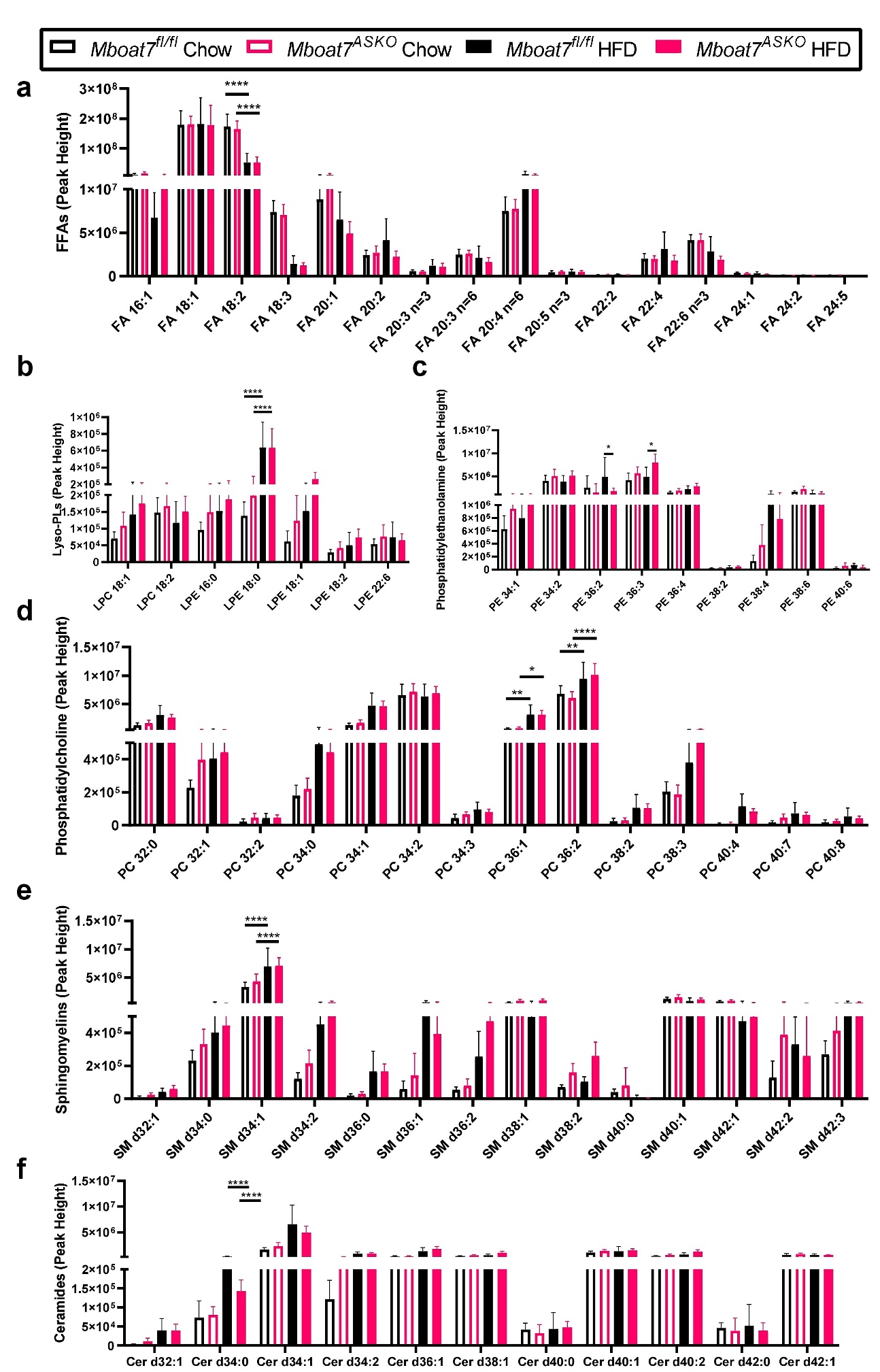
**

**Supplemental Fig. 7. Adipose Tissue Free Fatty Acids, Glycerophospholipids, Sphingomyelins, and Ceramides are Not Altered in *Mboat7^ASKO^* mice.** Male control (*Mboat7^fl/fl^*) or adipocyte-specific Mboat7 knockout mice (*Mboat7^ASKO^*) were fed chow or high fat diet (HFD) for 20-weeks. Gonadal adipose tissue (gWAT) free fatty acids (FFAs) (**a**), Lysophospholipids (LPLs) (**b**), Phosphatidyl-ethanolamines (PEs) (**c**), Phosphatidylcholines (PCs) (**d**), Sphingomyelins (SMs) (**e**), and Ceramides (Cers) (**f**) were quantified in *Mboat7^fl/fl^* or *Mboat7^ASKO^* mice were fed chow or HFD for 20-weeks via liquid chromatography mass spectrometry (n=5/group; Three-way ANOVA with Tukey’s *post-hoc* test). All data are presented as mean ± S.D.

**
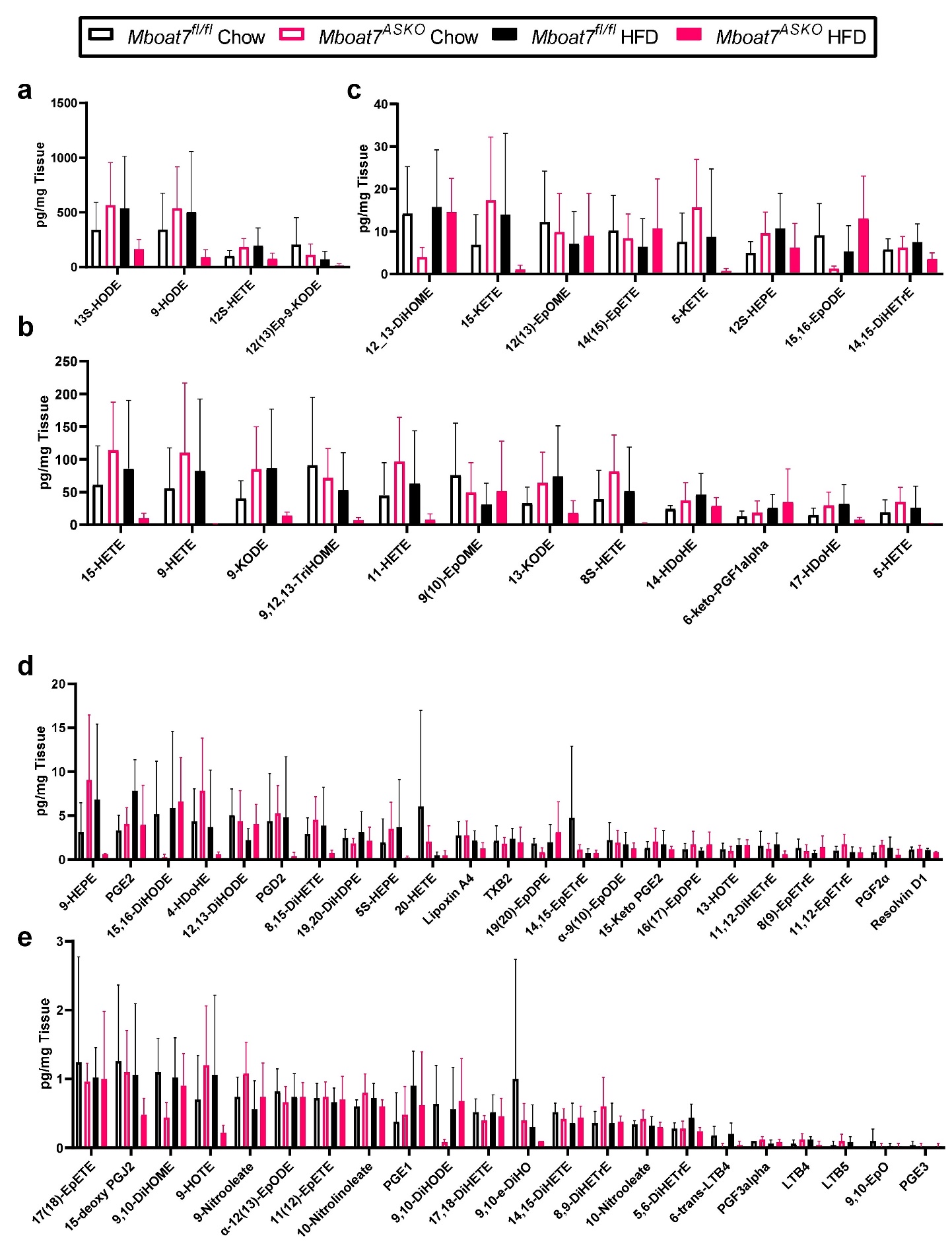
**

**Supplemental Fig. 8. Gonadal White Adipose tissue (gWAT) Oxylipins are Not Altered in *Mboat7^ASKO^* mice.** (**a-e**) Male control (*Mboat7^fl/fl^*) or adipocyte-specific Mboat7 knockout mice (*Mboat7^ASKO^*) were fed chow or high fat diet (HFD) for 20-weeks. Molecular species of oxylipins were quantified in gWAT of *Mboat7^fl/fl^* or *Mboat7^ASKO^* mice were fed chow or HFD for 20-weeks by liquid chromatography mass spectrometry (n=5/group); Three-way ANOVA with Tukey’s *post-hoc* test). All data are presented as mean ± S.D.

**
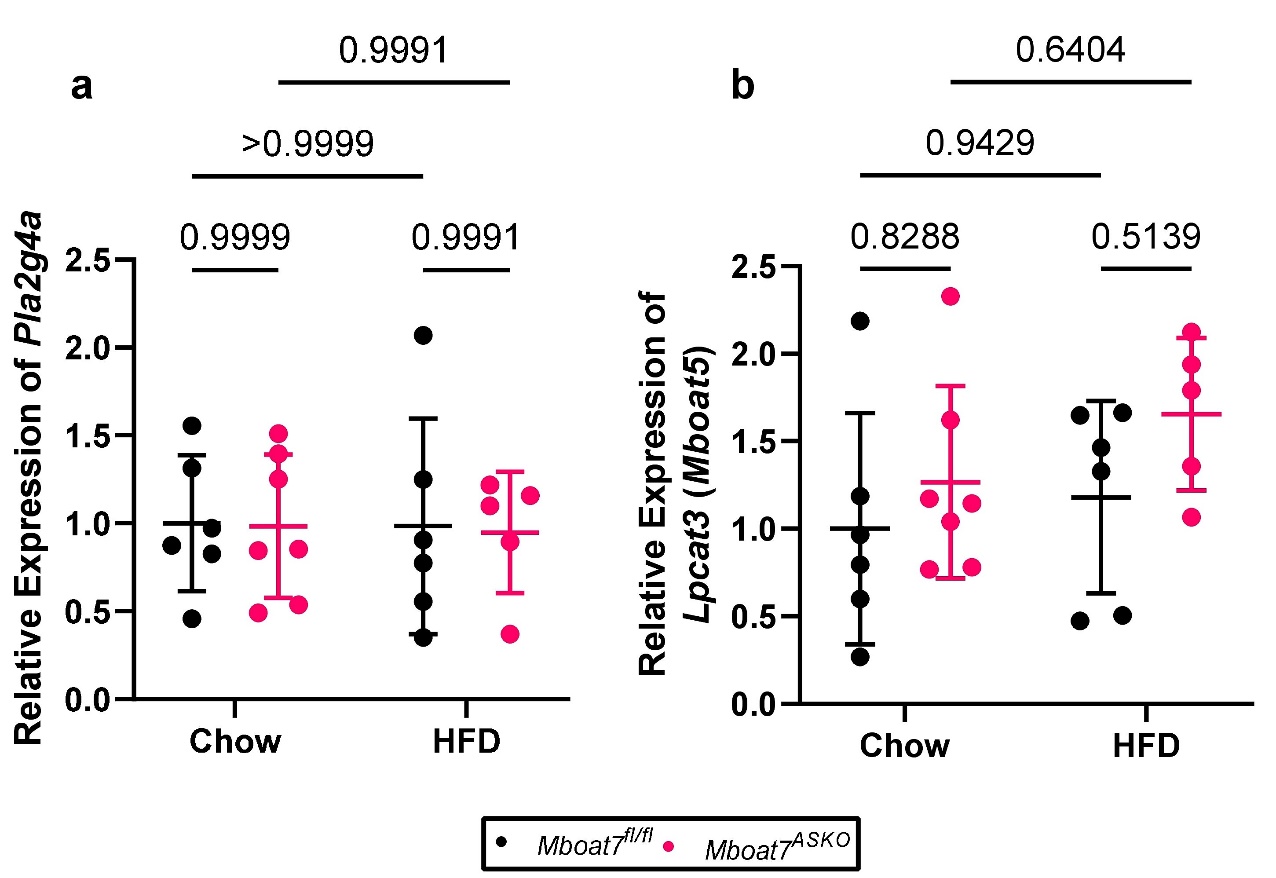
**

**Supplemental Fig. 9.** **Adipocyte-Specific *Mboat7* does not Alters *Pla2g4a* or *Lpcat3* expression in Adipose Tissue.** *Pla2g4a* (**a**) and *Lpcat3* (**b**) expression was measured in gWAT of Mboat7fl/fl or Mboat7ASKO mice fed chow or HFD for 20-weeks mice (n=5-7/group); Two-way ANOVA with Tukey’s *post-hoc* test). All data are presented as mean ± S.E.M.

**
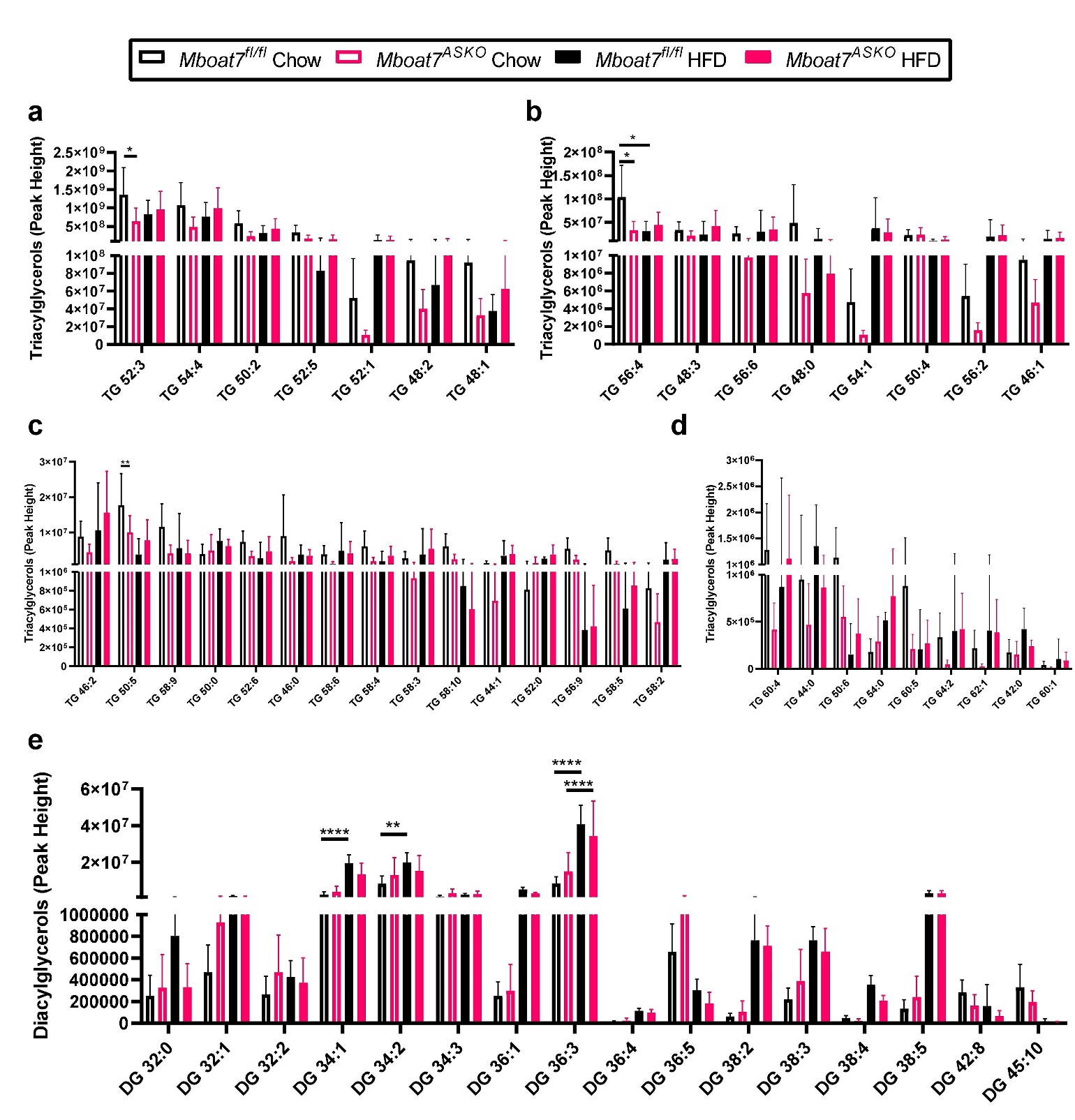
**

**Supplemental Fig. 10. Adipose tissue Diacylglycerol (DAG) and Triacylglycerol (TAG) are Not Altered in *Mboat7^ASKO^* Mice.** Male control (*Mboat7^fl/fl^*) or adipocyte-specific Mboat7 knockout mice (*Mboat7^ASKO^*) were fed chow or high fat diet (HFD) for 20-weeks. (**a-d**) Molecular species of triacylglycerols (TAG) were quantified via liquid chromatography mass spectrometry (n=5/group; Three-way ANOVA with Tukey’s *post-hoc* test). (**e**) Molecular species of diacylglycerols (DAG) were quantified in gWAT of *Mboat7^fl/fl^* or *Mboat7^ASKO^* mice were fed chow or HFD for 20-weeks via liquid chromatography mass spectrometry (n=5/group; Three-way ANOVA with Tukey’s *post-hoc* test). All data are presented as mean ± S.D.

**
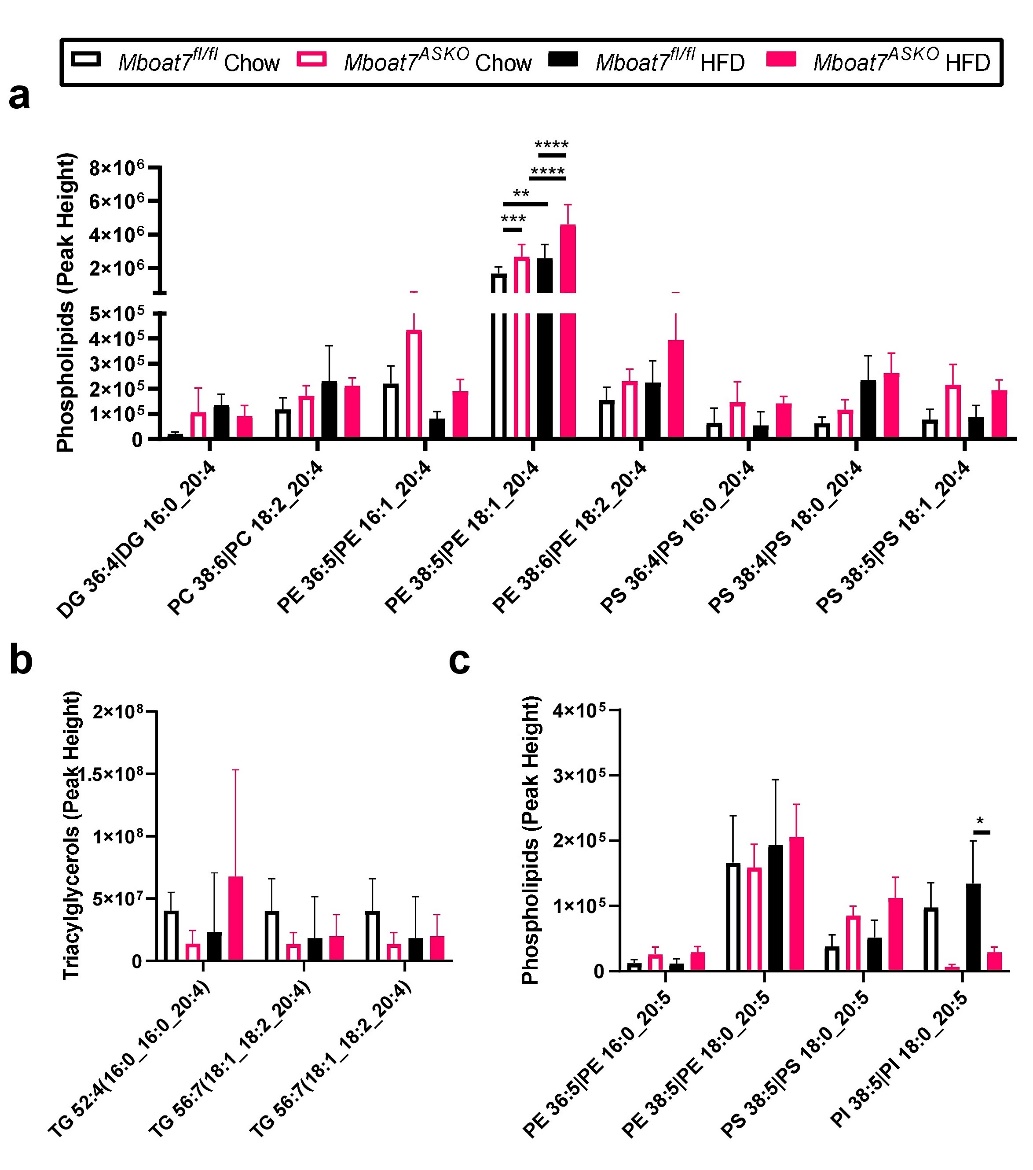
**

**Supplemental Fig. 11.** **Adipocyte-Specific *Mboat7* Deletion Alters Select Arachidonic Acid and Eicosapentaenoic Acid Containing Lipids in Adipose Tissue.** Male control (*Mboat7^fl/fl^*) or adipocyte-specific Mboat7 knockout mice (*Mboat7^ASKO^*) were fed chow or high fat diet (HFD) for 20-weeks. Phospholipids **(a,c)**, diacylglycerols (DAG) **(a)**, and triacylglycerols (TG) **(b)** containing arachidonic acid (20:4) were quantified in gWAT of *Mboat7^fl/fl^* or *Mboat7^ASKO^* mice were quantified by liquid chromatography mass spectrometry (n=5/group; Three-way ANOVA with Tukey’s *post-hoc* test). (**c**) Phospholipids containing eicosapentaenoic acid (20:5) were quantified in gWAT of *Mboat7^fl/fl^* or *Mboat7^ASKO^* mice were fed chow or HFD for 20-weeks by the West Coast Metabolomics’ complex lipid panel (n=5/group; Three-way ANOVA with Tukey’s *post-hoc* test). All data are presented as mean ± S.D.

**
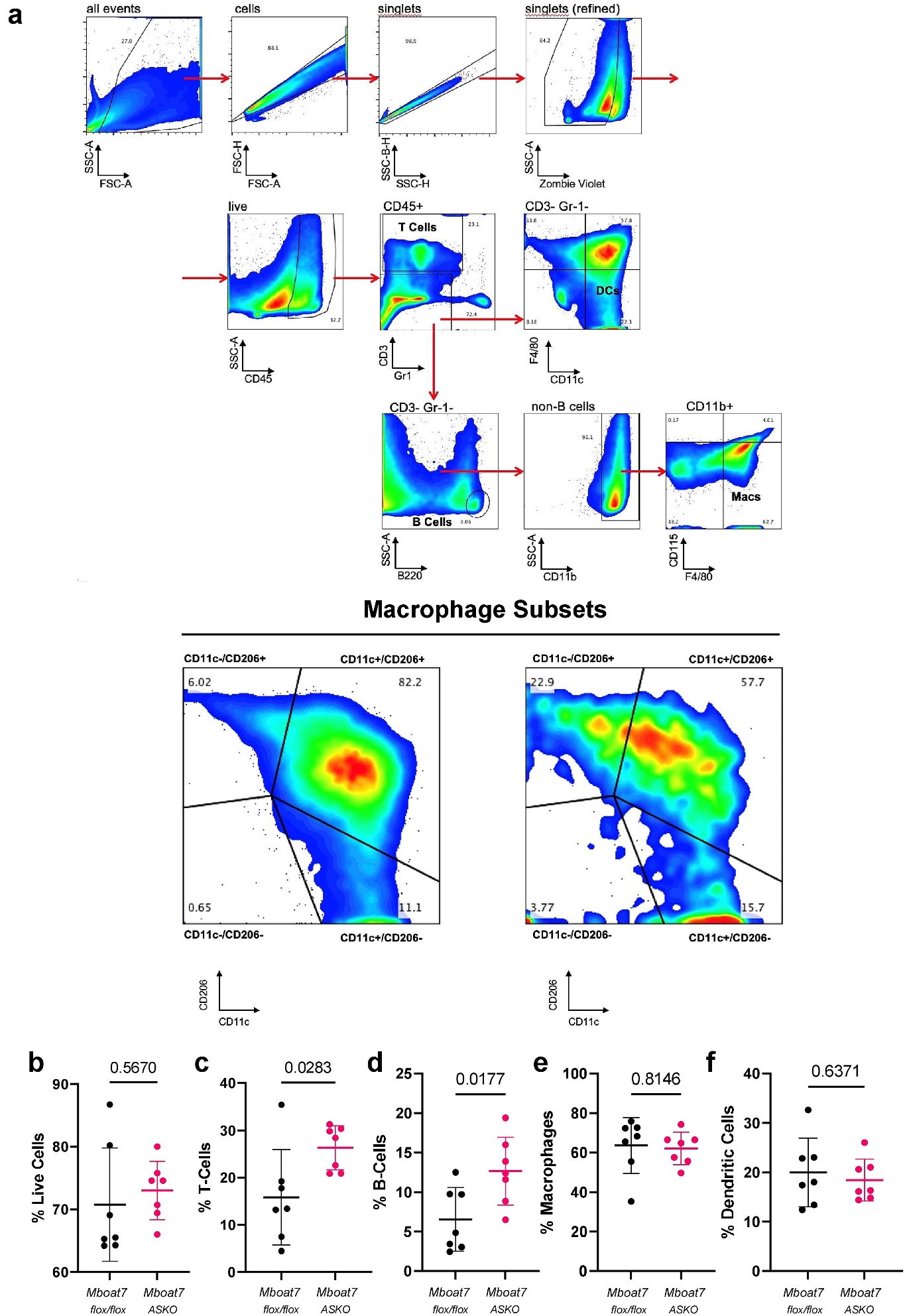
**

**Supplemental Fig. 12.** **Adipocyte-Specific *Mboat7* Deletion (*Mboat7^ASKO^*) Reorganizes White Adipose Tissue Immune Cell Populations.** Male control (*Mboat7^fl/fl^*) or adipocyte-specific Mboat7 knockout mice (*Mboat7^ASKO^*) were fed chow or high fat diet (HFD) for 20-weeks. The stromal vascular fraction of gonadal white adipose tissue (gWAT) was subjected to flow cytometric analysis of immune cell populations. **(a,b)** Gating strategy used for identifying subsets of adipose tissue macrophage populations. The percentage of live cells **(b)**, T-cells **(c)**, B-cells **(d)**, macrophages **(e)**, or dendritic cells **(f)** were quantified via flow cytometry.

**
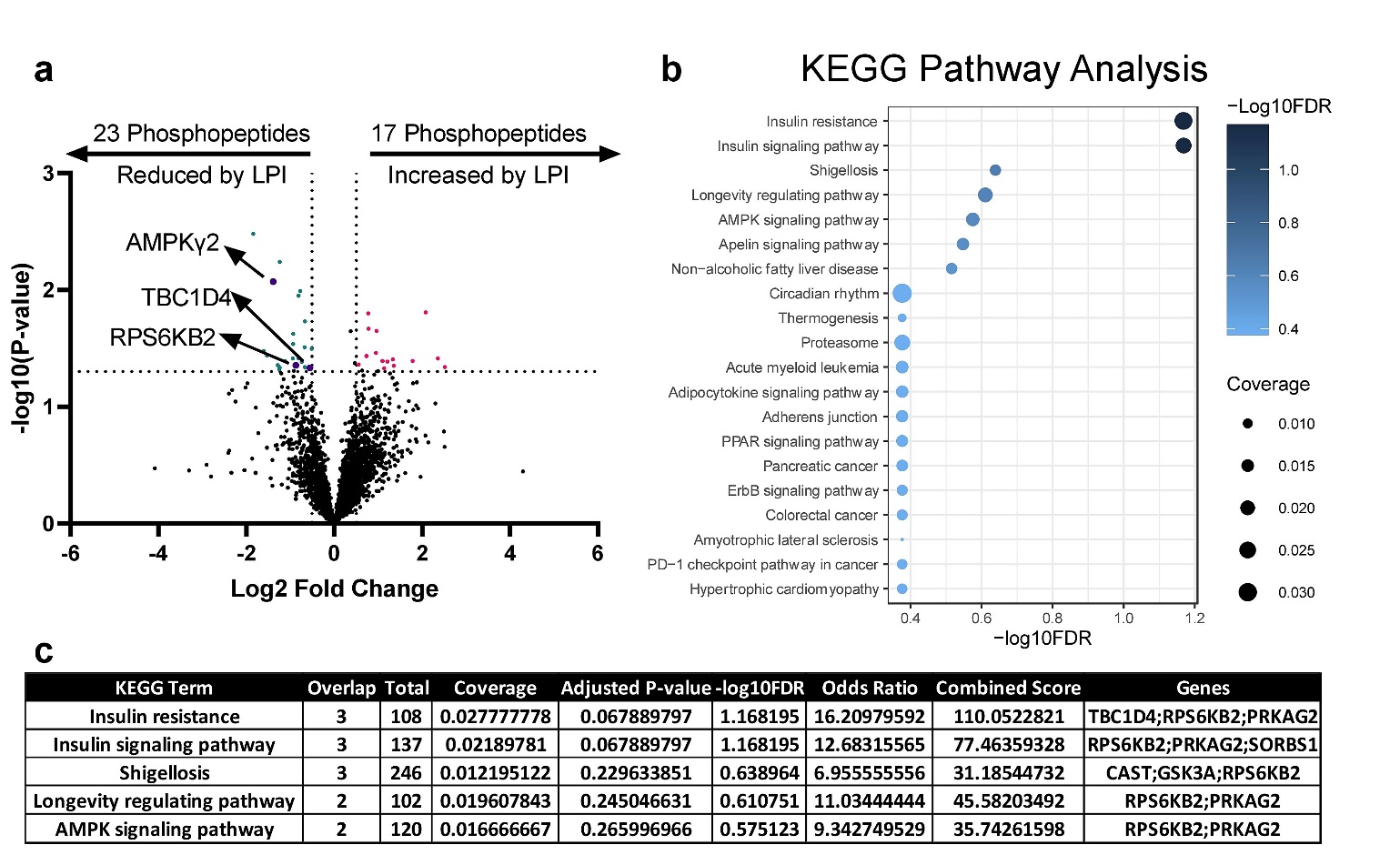
**

**Supplemental Fig. 13.** **LPI-18:1 does not significantly induce Phosphoproteomic signature associated with Insulin Resistance in gWAT of *Mboat7^flox/flox^* mice.** (**a**) Volcano plot of phospho-peptides upregulated and downregulated in gWAT of *Mboat7^flox/flox^* mice. Phospho-peptides determined to be |log2FoldChange|>0.5 different in the LPI and saline samples with a p-value < 0.05 (two-tailed t-test) were considered significantly differentially phosphorylated. (Average of n=4/group; pink dots represent significantly up-regulated phospho-peptides, green dots represent significantly down-regulated phospho-peptides; and dark purple dots represent insulin resistance associated phospho-peptides that are significantly different; light purple dots are used to highlight phospho-SREBP1 and phospho-FOXO1) (**b,c**) KEGG pathway analysis of significantly differentially phosphorylated peptides.

**Supplemental Table 1. Key reagents**

| **Key Resource Table** |  |  |  |  |  |
| --- | --- | --- | --- | --- | --- |
| Reagent type (species) or resource | Designation | Source or Reference | Identifiers | Additional Information | Research Resource Identifiers (RRID) |
| Genetic reagent (*M.musculus*) | *Mboat7tm1a(KOMP)Wtsi/Mboat7tm1a(KOMP)Wtsi* | PMID: 23472195 |  |  | RRID:MGI:5510874 |
| Genetic reagent (*M.musculus*) | *B6.FVB-Tg(Adipoq-cre)1Evdr/J* | Jackson Laboratory | Stock#: 028020 |  | RRID: IMSR_JAX:028020 |
| Genetic reagent (*M.musculus*) | *B6.Cg-Speer6-ps1Tg(Alb-cre)21Mgn/J* | Jackson Laboratory | Stock#: 003574 |  | RRID: IMSR_JAX:003574 |
| Antibody | Anti-MBOAT7 (Rat monoclonal) | PMID: 23097495 |  | 1:1000 | RRID: AB_2813851 |
| Antibody | Anti-Rat-HRP | Cell Signaling | Cat#: 7077 | 1:5000 | RRID: AB_10694715 |
| Antibody | Anti-GAPDH-HRP (Rabbit monoclonal) | Cell Signaling | Cat#: 8884 | 1:5000 | RRID: AB_11129865 |
| Antibody | BV510 Rat Anti-Mouse CD45 | BD Pharm | Cat#: 563891 | 1:100 | RRID:  AB_2734134 |
| Antibody | Anti-CD3e-APC-Cy7 (Hamster monoclonal) | BD Pharm | Cat#: 557596 | 1:100 | RRID:  AB_396759 |
| Antibody | Ly-6G/Ly-6C Monoclonal Antibody (RB6-8C5), FITC | Thermo | Cat#: 11-5931-82 | 1:100 | RRID:  AB_465314 |
| Antibody | CD45R (B220) Monoclonal Antibody (RA3-6B2), eFluor™ 506 | eBioscience | Cat#: 69-0452-82 | 1:100 | RRID:  AB_2637455 |
| Antibody | Anti-Cd11b-PE-Cy7  (Rat monoclonal) | BD Pharm | Cat#: 552850 | 1:100 | RRID:  AB_394491 |
| Antibody | PerCP/Cyanine5.5 anti-mouse F4/80 Antibody | Biolegend | Cat#:123128 | 1:100 | RRID:  AB_893484 |
| Antibody | CD115 (c-fms) Monoclonal Antibody (AFS98), PE | eBioscience | Cat#: 12-1152-83 | 1:100 | RRID:  AB_465809 |
| Antibody | Anti-Cd11c-Alexa  Fluor 700-(Hamster monoclonal) | BD Pharm | Cat#: 560583 | 1:100 | RRID:  AB_1727421 |
| Antibody | Anti-CD206 Alexa  Fluor 647 (Rat monoclonal) | Biolegend | Cat#: 141712; | 1:100 | RRID: AB_10900420 |
| Commercial assay kit | ALT kit | Sekisui Diagnostics | 318-30 |  |  |
| Commercial assay kit | Liver TG | Wako | 994-02891 |  |  |
| Commercial assay kit | Free Cholesterol | Wako | 993-02501 |  |  |
| Commercial assay kit | Total Cholesterol | Fisher Sci | TR134321 |  |  |
| Commercial assay kit | Metabolic Hormone Combo 1 | MesoScale Discovery | Cat#: K15306K |  |  |
| Commercial assay kit | NEFA Kit | Wako | 999-34691, 995-34791, 991-34891, 993-35191, 276-76491 |  |  |
| Commercial assay kit | Glycerol kit | Cayman Chemical | 10010755 |  |  |
| Commercial assay kit | C-Peptide ELISA | Crystal Chem | 90050 |  |  |
| Chemical compound, drug | Βeta-3 AR Agonist | Sigma | C5976-5mg |  |  |
| Chemical compound, drug | Glucose | Sigma | G8270 |  |  |
| Chemical compound, drug | Insulin (HumalinR) | Lily | 0002-8215-17 |  |  |
| Chemical compound, drug | LPI (18:1) | Avanti | 850100P |  |  |
| Sequence Based Reagent | Cyclophilin A Fwd | Sigma | 5’-GCGGCAGGTCCATCTACG-3’ |  |  |
| Sequence Based Reagent | Cyclophilin A Rev | Sigma | 5’-GCCATCCAGCCATTCAGTC-3’ |  |  |
| Sequence Based Reagent | Pla2g4a Fwd | Sigma | 5’-GTGGGAGAGAAGAAAGAAGTCC-3’ |  |  |
| Sequence Based Reagent | Pla2g4a Rev | Sigma | 5’-GTAGGTCTGGGCATGAACAA-3’ |  |  |
| Sequence Based Reagent | Lpcat3 Fwd | Sigma | 5’-TCGTGCTTCAGTTCCTCATC-3’ |  |  |
| Sequence Based Reagent | Lpcat3 Rev | Sigma | 5’-CCGGTGGCTGTGTACTAATATC-3’ |  |  |
